# Health effects of the Brazilian Conditional Cash Transfer programme over 20 years and projections to 2030: a retrospective analysis and modelling study

**DOI:** 10.1016/S2468-2667(25)00091-X

**Published:** 2025-05-29

**Authors:** Daniella Medeiros Cavalcanti, José Alejandro Ordoñez, Andrea Ferreira da Silva, Elisa Landin Basterra, Ana L Moncayo, Carlos Chivardi, Philipp Hessel, Alberto Pietro Sironi, Rômulo Paes de Sousa, Tereza Campello, Luis Eugênio Souza, Davide Rasella

**Affiliations:** a Institute of Collective Health, Federal University of Bahia, Bahia, Brazil; b Pontificia Universidad Católica de Chile, Santiago, Chile; c Centro de Investigación para la Salud en América Latina (CISeAL), Pontificia Universidad Católica del Ecuador, Quito, Ecuador; d Centre for Health Economics, University of York, York, UK; e Alberto Lleras Camargo School of Government, Universidad de los Andes, Bogotá, Colombia; f René Rachou Institute at Fiocruz, Belo Horizonte, Brazil; g Brazilian Development Bank (BNDES), Rio de Janeiro, Brazil; h Instituto de Salud Global (ISGlobal), Hospital Clinic-Universitat de Barcelona, Barcelona, Spain; i Institución Catalana de Investigación y Estudios Avanzados (ICREA), Pg Lluís Companys 23, Barcelona, Spain

## Abstract

**Background:**

In 2024, Brazil celebrated the 20th anniversary of the Bolsa Família Program (BFP), one of the world's oldest and largest conditional cash transfer (CCT) programmes, covering more than 50 million Brazilians. This study aimed to evaluate the effect of the BFP on overall mortality and hospitalisation rates over the past two decades, and to forecast the potential effects of expanding this programme until 2030.

**Methods:**

This study combined retrospective impact evaluations in Brazil from 2000–19 with microsimulation models up to 2030. First, the effect of the BFP on overall mortality and hospitalisation rates was estimated across different age groups, adjusting for all relevant demographic, socioeconomic, and health-care factors. Fixed-effect multivariable Poisson models were then applied to 3671 municipalities with adequate quality vital statistics data. The three exposure variables of BFP were target coverage, benefits adequacy (average transfer per family), and the interaction of coverage and adequacy. Several sensitivity and triangulation analyses were conducted, including difference-in-difference models with propensity-score matching. Previous longitudinal datasets were then integrated with validated dynamic microsimulation models to project trends up to 2030.

**Findings:**

High coverage of BFP was associated with a significant reduction in overall age-standardised mortality rates (rate ratio [RR] 0·824 [95% CI 0·807–0·842]). High adequacy of BFP was associated with a reduction in overall age-standardised mortality (0·849 [0·833–0·866]). Our models estimated that the BFP prevented 8 225 390 (95% CI 8 192 730–8 257 014) hospitalisations and 713 083 (702 949–723 310) deaths in 2000–19. Stronger effects were found in BFP high coverage and high adequacy scenario, resulting in large reductions in under-5 mortality (RR 0·67 [95% CI 0·65–0·69]) and hospitalisation of individuals older than 70 years (0·52 [0·50–0·53]). Expanding BFP coverage could avert an additional 8 046 079 (95% CI 8 023 306–8 068 416) hospitalisations and 683 721 (676 494–690 843) deaths by 2030, compared with scenarios of reduced coverage.

**Interpretation:**

CCT programmes have strongly contributed to the reduction of morbidity and mortality in Brazil, having prevented millions of hospitalisations and deaths in the past two decades. During the current period of polycrisis, the expansion of CCTs in terms of coverage and benefits could prevent a large number of hospitalisations and deaths worldwide, and should be considered a crucial strategy for achieving the UN health-related Sustainable Development Goal 3.

**Funding:**

UK Foreign, Commonwealth and Development Office, UK Medical Research Council, and the Wellcome Trust (grant number MC_PC_MR/T023678/1).

**Translation:**

For the Portuguese translation of the summary see Supplementary Materials section.

## Introduction

2024 marked the 20th anniversary of the Bolsa Família Program (BFP) in Brazil,^1^ one of the world's pioneering and largest conditional cash transfer (CCT) programmes.^2^, ^3^ CCT programmes transfer cash to poor households on the condition that parents meet specific requirements (named conditionalities), focused on health and education, aimed at alleviating short-term poverty while breaking the intergenerational cycle of poverty.^4^ In the case of BFP, eligibility is determined by per capita household income: families in poverty, if they have children (aged <7 years), pregnant women, or adolescents (aged <18 years) qualify for benefits. Conditionalities include school attendance, vaccinations, and prenatal care, reinforcing the role of BFP in improving human capital.^1^

Currently, the BFP covers more than 20 million families (55·1 million people), transferring an average of US$139 to each household monthly, with an overall budget of approximately US$34·5 billion in 2023. Since its inception in 2004, the BFP has had substantial effects on poverty reduction and educational indicators,^2^, ^3^, ^5^, ^6^ directly improving the quality of life of beneficiary families.

**Research in context**

**Evidence before this study**

To investigate the available evidence on the effects of conditional cash transfer (CCT) programmes on overall hospitalisation and mortality, we initially searched PubMed for studies published containing the following terms: “cash transfers” [MeSH Terms] OR “conditional cash transfer” [MeSH Terms] AND “mortality” OR “hospitalization”. The search dates were from database inception until Nov 30, 2024. There were no restrictions on language of publication. We also checked the referenced studies of the selected articles.

Our search found several articles on CCTs, some of which were associated with health outcomes. Studies from various geographical regions and both high-income and low-income and middle-income countries (LMICs) were included. Previous studies indicated mixed results. Some studies suggested that cash transfers, particularly CCTs, are associated with improved health outcomes, including reduced hospitalisation rates and mortality, particularly in vulnerable populations, such as children and older adults. However, the evidence is heterogeneous, with some studies showing minimal or no significant impact. In terms of the Bolsa Família Program, previous studies investigated the effects of this CCT on specific diseases, such as tuberculosis, HIV/AIDS, and malnutrition. Other studies found effects on specific age groups, such as child or infant morbidity or mortality, especially through the mechanism of vaccination and nutritional monitoring, and poverty-related diseases, such as diarrhoea and malnutrition. All assessed studies evaluated CCT over relatively short periods (ie, up to 8 years), focusing on specific health outcomes and in specific age groups.

**Added value of this study**

To the best of our knowledge, this is the first study that used a robust analytical approach over a 20-year period to comprehensively evaluate the effects of one of the world's largest CCTs on hospitalisation and mortality rates, overall and stratified by age group. This study uses the datasets and parameters of the retrospective impact evaluations to develop forecasting analyses, comparing the effects of alternative policy implementation scenarios on overall hospitalisation and mortality up to 2030, the last year of SDGs. Moreover, it is the first study that uses different measures of CCT implementation, including coverage, adequacy, and the combination of both, to estimate the prevented burden of hospitalisation and mortality over the past two decades, as well as in the coming decade.

**Implications of all the available evidence**

Our analyses show that the implementation of a nationwide CCT programme in an LMIC can strongly contribute to the reduction of hospitalisation and mortality rates, potentially averting millions of deaths and hospitalisations, and making important contributions to the achievement of health-related SDGs.

Although the BFP, and many other CCT programmes worldwide, have been conceptualised and developed mainly to reduce poverty and socioeconomic inequalities, they have also had unexpectedly strong effects on many health outcomes, not only in children, but also in the adults living in the beneficiary families.^7^, ^8^ Therefore, CCT programmes can serve as crucial policies not only for the achievement of UN Sustainable Development Goal (SDG) 1, poverty eradication, but also for advancements in SDG 3, good health and wellbeing.

These co-benefits are particularly important considering that the consequences of the COVID-19 pandemic, climate change, and recent conflicts (the so-called three Cs) have caused substantial setbacks to global poverty and global health.^9^ There have been repeated calls worldwide to expand poverty-reduction interventions as potential mitigation policies, increasing the number of benefits, and including the newly impoverished families among the beneficiaries. Conversely, the rise in public debt in most low-income and middle-income countries (LMICs), following the COVID-19 pandemic, could lead to the implementation of fiscal austerity policies, resulting in budget cuts for social protection and health-care systems.^9^, ^10^ Despite the importance of maintaining the financial balance of public accounts, continuing with social programmes, such as the BFP, should be considered paramount to reduce poverty and social inequalities and promote population health.

In this context, the Global Alliance Against Hunger and Poverty (GAAHP) was created by the Group of Twenty (G20) countries in 2024; its mission underscores the significance of social protection measures, such as the BFP, in tackling global poverty and inequality. By prioritising inclusive growth and poverty reduction, the GAAHP highlights income transfer programmes as essential tools to mitigate the economic impacts of crises, enhance resilience, and promote social stability. Moreover, the GAAHP advocates for innovative financing mechanisms and strengthened international cooperation to support these programmes, emphasising the urgent need to sustain and expand them—particularly in LMICs—to address economic instability and climate change challenges.

A substantial body of research has evaluated the effects of CCT programmes on the use of health services, nutritional status, and a wide range of health outcomes.^11^, ^12^, ^13^, ^14^ However, only a few studies have evaluated the effects of CCT programmes on country-wide mortality and hospitalisation rates,^7^, ^15^, ^16^ and none have analysed the effects of different characteristics of their implementation and coverage over decades, estimating the prevented burden of disease and mortality, and forecasting their future impact.

This study aims to evaluate the effects of the first 20 years of the nationwide expansion of the Brazilian CCT programme, both in terms of coverage and benefits, on overall hospitalisation and mortality rates, and to forecast the health effects of alternative implementation scenarios up to 2030, the target year for the UN SDGs.

## Methods

### Study design

This study integrated a retrospective impact evaluation with forecasting analyses. The retrospective impact evaluation had a longitudinal ecological design, whereby municipalities (unit of analysis) were observed over time. This longitudinal dataset combined aggregated health, socioeconomic, and BFP data from several sources (all data used in this study are publicly available; appendix 2 p 6) from 2000–23. From the total of 5570 municipalities in Brazil, we selected a subset of 3671 municipalities with adequate quality of civil registration and vital statistics, as in previous studies with similar methodologies,^2^, ^17^, ^18^ according to a validated multidimensional criterion that considered the age-standardised mortality rate of the municipality, the ratio between registered and estimated birth rates, the percentage of poorly defined deaths, and the mean deviation of all the previous parameters (appendix 2 pp 9–10).^17^ Although the exclusion of municipalities with an inadequate level of vital information could reduce the external validity of the findings, it is considered an essential factor for strengthening the internal validity of the study and reducing any possible bias due to changes in the quality of the death notification system—mainly reduction of sub-notifications—during the study period.^17^

Models considering all Brazilian municipalities and models with a weighting based on the municipal population were also estimated (appendix 2 p 21). Age-standardised all-cause mortality and hospitalisation rates were calculated for the entire population and used as dependent variables. We also included analyses by major age groups as secondary dependent variables: younger than 5 years, 5–69 years, and 70 years and older. Complementary analyses by smaller age subgroups can be found in appendix 2 (pp 28–29).

The coverage of BFP was calculated, similarly to previous studies,^2^, ^3^, ^5^ as the number of families enrolled in the BFP in a municipality divided by the number of eligible families (according to BFP criteria) in the same municipality (ie, the target coverage).^2^ We also calculated the adequacy of BFP benefits as the total amount of money transferred to all families divided by the number of families enrolled in the BFP in a municipality. As in previous studies,^2^, ^19^, ^20^ we categorised BFP coverage and adequacy to estimate the dose–response effect related to increasing degrees of implementation of the interventions. Using previously established reference thresholds,^2^, ^18^, ^20^ we created four levels of BFP target coverage: low (0–29·9%), intermediate (30·0–69·9%), high (70·0–99·9%), and consolidated (100%). In the absence of reference values from the literature, adequacy was categorised using quartiles: low (0–24·9 percentile), intermediate (25·0–49·9 percentile), high (50·0–74·9), and consolidated (75·0–100·0 percentile).

All relevant time-variant demographic, socioeconomic, and health-care-adjusting variables, according to the literature,^2^, ^19^, ^20^, ^21^ were included in the models: poverty rate, illiteracy rate, Gini index, urbanisation rate, fertility rate, percentage of households with inadequate sanitation, percentage of households with piped water, number of physicians per 1000 individuals, number of hospital beds per 1000 individuals, and the coverage of other social programmes, such as social pensions (Benefício de Prestação Continuada) and primary health care (Estratégia de Saúde da Família). A wide range of other additional covariates was also tested in a sensitivity analysis (appendix 2 pp 15–16, 30). As in previous studies,^2^, ^3^, ^5^ we dichotomised these covariates according to their median value over the period.^2^, ^3^, ^5^, ^18^, ^22^ We included time dummy variables (for 2008–09, 2013–14, and 2015–16) to adjust for major economic shocks that occurred in Brazil in the past two decades.^21^, ^22^

### Data sources

The data on the number of deaths, hospitalisations, beds, and physicians were collected from the Brazilian Ministry of Health. The number of beneficiaries of the BFP and the total amount transferred per family were collected from the Brazilian Ministry of Social Development, and socioeconomic and demographic variables were obtained from surveys and censuses conducted by the Brazilian Institute of Geography and Statistics. The complete list of data sources and related detailed methods are available in appendix 2 (p 6).

### Statistical analysis

The effect of BFP target coverage and BFP adequacy on overall mortality and hospitalisation in 2000–19 was measured using Poisson multivariable regression models with fixed-effects specifications. This consolidated methodological approach evaluates the effects of nationwide interventions on hospitalisation and mortality rates with aggregate-level panel data.^2^, ^18^, ^23^ Fixed-effects models include a term to control for unobserved characteristics of the unit of analysis that are approximately constant during the study period (eg, some geographical, historical, or sociocultural aspects of each municipality), which were not included in the model as confounding variables and could be associated both with the outcome and with the implementation of the intervention.^24^ The Poisson distribution with robust standard errors for heteroscedasticity and serial correlation is used to deal with the overdispersion of mortality data in the municipalities.^23^ To evaluate the robustness of the estimates, several sensitivity analyses were carried out (appendix 2 pp 13–31). First, the models were fitted with continuous variables and changing variable thresholds to evaluate the influence of the categorisation. Second, the models were fitted with all 5570 municipalities in Brazil (ie, including municipalities with inadequate quality of civil registration and vital statistics) to assess the external validity of resultant estimates. Third, different sets of time variables were tested to investigate the influence and relevance of the time dummies. Fourth, negative binomial regression models were fitted and resultant estimates were compared with those of negative binomial models, in order to evaluate the stability of the results with alternative models. Fifth, the BFP effects on overall mortality rates due to external causes were estimated as an outcome for use as a negative control as they should not be affected by BFP.^2^, ^5^, ^21^ Finally, to have a high degree of confidence in the causal inference and the overall impact evaluation, additional triangulation analyses^25^ were performed, using difference-in-difference with propensity score matching,^26^ evaluating the municipalities with low BFP coverage versus medium and high coverage in the years 2004 and 2019. Moreover, we evaluated all sensitivity, triangulation, and complementary results according to consolidated causal inference criteria (appendix 2 pp 13–31).^25^ We used Monte Carlo simulations to estimate the number of hospitalisations and deaths averted by CCT programmes in 2000–19, comparing predicted outcomes to a counterfactual scenario without the programme (ie, no CCT coverage) and performing 10 000 iterations to ensure estimate stability. We used Stata (version 17.0) for database processing and analysis.

We used validated municipal-level microsimulation models to forecast the effects of potential BFP expansions or reductions on health outcomes until 2030. Microsimulation is considered to be one of the most accurate forecasting methods because it allows for modelling municipality-specific characteristics and their associated outcome probabilities, in particular when developed as projections from the existing retrospective real-data cohorts, maintaining their original variable distribution, variable correlations, and municipal-specific trends.^26^ Our modelling approach, based on previous studies,^18^, ^22^, ^27^ was conducted in two stages. In stage 1, a synthetic cohort of all Brazilian municipalities for 2024–30 was created, which was extrapolated from and modelled on each municipal-level independent variable from the 2000–23 dataset. In stage 2, mortality and hospitalisation rates were predicted, using these independent variables as inputs in the same multivariate regression models used in the retrospective analysis, including estimates of their effects.

In stage 1, adjustments to the BFP eligibility criteria were simulated, with a focus on increasing the monetary threshold that determines family eligibility based on monetary poverty. Currently, the BFP eligibility criteria are nearly aligned with the World Bank's extreme poverty line of US$2·15 per day. Therefore, poverty thresholds based on half or a quarter of the Brazilian minimum wage (ie, BRL$1412 per month, equivalent to approximately US$291 in January, 2024, when the wage was established) were simulated (appendix 2 pp 33–34).

Regarding the policy response to this increase in the number of eligible families, three changes in BFP coverage were simulated: the expansion scenario (increasing coverage to include 100% of families with incomes below half of the minimum wage); the maintenance scenario (maintaining current coverage levels); and the severe fiscal austerity scenario (decreasing BFP coverage). The severe fiscal austerity scenario was derived from a validated model already used in previous studies,^22^, ^27^ which projected the effects of the current fiscal austerity measures on the coverage of the BFP (appendix 2 pp 33–34). The projection is proportional to the reduction of government expenditure on social protection observed from 2014–19.^28^ For each outcome and each scenario, 10 000 Monte Carlo simulations were performed, allowing parameter values to vary in each simulation cycle according to their assumed underlying distribution. Further details of the modelling process, following the international model reporting guidelines (ISPOR-SMSM),^29^ including calibration of models, internal and external validation, parameter distributions for Monte Carlo simulations, and model equations are provided in appendix 2 (pp 33–36). For the forecasting analyses, we used R (version 4.1.2).

### Role of the funding source

The funders had no role in the study design, data collection, data analysis, data interpretation, or writing of the manuscript and the decision to submit.

## Results

In 2004–19, the mean age-standardised mortality rate of the 3671 municipalities studied decreased by 1·92% (table 1), with the strongest reduction in children younger than 5 years (–10·47%) and the least reduction in individuals aged 5–69 years (–0·48%). In the same period, the average age-standardised hospitalisation rates decreased by 0·62%. The target coverage of the BFP almost doubled between 2004 and 2019 (48·58%), whereas the coverage of the Estratégia de Saúde da Família increased by 46·80% and that of the Benefício de Prestação Continuada by 1·21%. Overall, socioeconomic, health-care, and living conditions improved during the study period.

**Table 1:** Mean rates of municipal mortality, conditional cash transfer coverage, and other variables for selected municipalities of Brazil from 2004 to 2019

		**Year**			**Change in rate (2004–19)**	
		2004	2010	2019	Absolute	Relative
**Mortality rate for age group (per 1000 individuals)**						
Overall		7·57 (1·44)	6·29 (0·87)	5·65 (0·79)	−1·92	−25·36
	<5 years (per 1000 livebirths)	24·32 (15·10)	15·62 (6·82)	13·85 (5·78)	−10·47	−43·05
	5–69 years	3·84 (0·89)	3·56 (0·61)	3·36 (0·57)	−0·48	−12·50
	≥70 years	68·03 (15·87)	56·47 (8·93)	52·34 (7·42)	−15·69	−23·06
**Hospitalisation rate for age group (per 1000 individuals)**						
Overall		2·00 (6·03)	1·47 (2·31)	1·38 (3·58)	−0·62	−31·00
	<5 years (per 1000 livebirths)	799·30 (1484·37)	705·38 (1416·78)	644·14 (1320·49)	−155·16	−19·41
	5–69 years	4·12 (12·48)	2·69 (6·66)	2·90 (7·63)	−1·23	−29·61
	≥70 years	185·30 (276·42)	106·62 (149·83)	66·60 (95·12)	−118·70	−64·06
**Bolsa Família Program**						
Coverage of all population (%)		7·86 (7·70)	19·07 (14·63)	16·78 (14·14)	8·92	113·74
Coverage of target population (%)		50·67 (19·49)	98·75 (4·38)	99·25 (5·25)	48·58	95·88
Adequacy (BRL$)*		71·67 (6·57)	126·85 (12·29)	408·05 (52·21)	336·38	469·35
**Other social programmes**						
Benefício de Prestação Continuada coverage (%)		1·02 (0·64)	1·74 (0·95)	2·23 (1·11)	1·21	118·63
Estratégia de Saúde da Família coverage (%)		13·01 (21·76)	47·33 (31·20)	59·81 (27·71)	46·80	359·72
**Other covariates**						
Fertility rate (births per woman)		3·33 (0·67)	2·89 (0·49)	2·55 (0·38)	−0·78	−23·42
Poverty rate (%)		23·07 (17·85)	11·73 (11·79)	7·48 (9·23)	−15·59	−67·58
Proportion of individuals older than 15 years who are illiterate (%)		10·89 (8·93)	7·60 (6·72)	4·73 (5·02)	−6·16	−56·57
Gini index		56·70 (5·83)	53·17 (6·88)	52·43 (9·70)	−4·27	−7·53
Piped water coverage (%)		80·39 (20·45)	84·97 (16·86)	87·44 (15·90)	7·06	8·77
Adequate sanitation coverage (%)		11·97 (12·66)	22·50 (18·52)	27·28 (22·03)	15·31	127·90
Urbanisation rate (%)		86·71 (18·00)	88·96 (16·20)	90·66 (14·96)	3·95	4·56
Hospital bed rate (per 1000 individuals)		2·93 (2·04)	2·56 (1·63)	2·24 (1·43)	−0·69	−23·55
Physician rate (per 1000 individuals)		1·41 (0·93)	1·69 (1·13)	2·16 (1·46)	0·75	53·19

Data are mean (SD). Absolute change refers to the difference in values between two timepoints, whereas the relative change refers to the growth rate or percentage variation over time. The total number of municipalities included is 3671, which represents a subset of the total 5570 municipalities in Brazil.

* The adequacy of the programme was calculated by dividing the total amount of money transferred to all families by the number of families enrolled in the BFP in a municipality.

Table 2 shows the adjusted associations of the overall mortality and hospitalisation rates with the coverage and adequacy levels of the BFP. Consolidated coverage of the BFP was associated with a statistically significant reduction in overall age-standardised mortality, with a rate ratio (RR) of 0·824 (95% CI 0·807–0·842). Consolidated adequacy of BFP was also associated with a statistically significant reduction of overall age-standardised mortality rates (RR 0·849 [0·833–0·866]). When combining these two measures of BFP, we found that municipalities with high coverage and high adequacy substantially reduced rates of overall age-standardised hospitalisation (RR 0·775 [0·756–0·795]) and mortality (RR 0·723 [0·711–0·735]). The low coverage and high adequacy group presented stronger effects in mortality rate reduction (0·815; 0·804–0·825) compared with the high coverage and low adequacy group (0·920; 0·911–0·929).

**Table 2:** RRs from the fixed-effect Poisson models for the association between age-standardised hospitalisation and mortality rates with the Bolsa Família Program coverage and adequacy

		**Age-standardised hospitalisation RR**			**Age-standardised mortality RR**		
		Coverage	Adequacy	Adequacy × coverage	Coverage	Adequacy	Adequacy × coverage
Bolsa Família Program target population coverage							
	Low (0–29·9%)	1 (ref)	..	..	1 (ref)	..	..
	Intermediate (30–69·9%)	0·884 (0·874–0·893; p<0·01)	..	..	0·924 (0·912–0·937; p<0·01)	..	..
	High (70–99·9%)	0·857 (0·847–0·867; p<0·01)	..	..	0·890 (0·880–0·899; p<0·01)	..	..
	Consolidated (100%)	0·789 (0·779–0·799; p<0·01)	..	..	0·824 (0·807–0·842; p<0·01)	..	..
Bolsa Família Program adequacy							
	Low (<BRL$61·44)	..	1 (ref)	..	..	1 (ref)	..
	Intermediate (≥BRL$61·44 to <BRL$99·13)	..	0·852 (0·843–0·861; p<0·01)	..	..	0·900 (0·888–0·913; p<0·01)	..
	High (≥BRL$99·13 to <BRL$151·23)	..	0·884 (0·877–0·892; p<0·01)	..	..	0·852 (0·834–0·871; p<0·01)	..
	Consolidated (≥BRL$151·23)	..	0·844 (0·835–0·853; p<0·01)	..	..	0·849 (0·833–0·866; p<0·01)	..
Bolsa Família Program adequacy × target coverage							
	Low adequacy × low coverage	..	..	1 (ref)	..	..	1 (ref)
	Low adequacy × high coverage	..	..	0·949 (0·941–0·957; p<0·01)	..	..	0·920 (0·911–0·929; p<0·01)
	High adequacy × low coverage	..	..	0·866 (0·853–0·879; p<0·01)	..	..	0·815 (0·804–0·825; p<0·01)
	High adequacy × high coverage	..	..	0·775 (0·756–0·795; p<0·01)	..	..	0·723 (0·711–0·735; p<0·01)
Control variables*							
	Others social programmes	0·997 (0·983–1·012; p>0·1)	1·024 (1·008–1·040; p<0·01)	0·966 (0·957–0·975; p<0·01)	0·972 (0·964–0·981; p<0·01)	0·986 (0·978–0·995; p<0·01)	0·985 (0·971–1·000; p<0·05)
	Fertility rate	1·018 (1·003–1·034; p<0·05)	1·028 (1·012–1·045; p<0·01)	0·986 (0·976–0·996; p<0·01)	0·999 (0·989–1·008; p>0·1)	1·005 (0·993–1·018; p>0·1)	0·997 (0·981–1·013; p>0·1)
	Poverty rate	0·956 (0·940–0·973; p<0·01)	1·01 (0·994–1·026; p>0·1)	0·967 (0·955–0·980; p<0·01)	0·966 (0·953–0·979; p<0·01)	1·012 (0·999–1·026; p<0·1)	0·953 (0·937–0·969; p<0·01)
	Proportion of individuals older than 15 years who are illiterate	1·026 (1·005–1·047; p<0·05)	1·025 (1·004–1·046; p<0·05)	0·981 (0·967–0·994; p<0·01)	0·988 (0·975–1·002; p>0·1)	0·979 (0·965–0·994; p<0·01)	1·009 (0·989–1·030; p>0·1)
	Gini Index	1·028 (1·014–1·042; p<0·01)	1·041 (1·027–1·055; p<0·01)	1·005 (0·994–1·016; p>0·1)	1·027 (1·017–1·038; p<0·01)	1·030 (1·019–1·042; p<0·01)	1·01 (0·996–1·023; p>0·1)
	Piped water	0·988 (0·970–1·005; p>0·1)	0·972 (0·955–0·990; p<0·01)	1·032 (1·022–1·043; p<0·01)	1·027 (1·015–1·039; p<0·01)	1·023 (1·013–1·032; p<0·01)	1·003 (0·986–1·021; p>0·1)
	Households with inadequate sanitation	0·898 (0·881–0·916; p<0·01)	0·886 (0·869–0·903; p<0·01)	1·035 (1·021–1·049; p<0·01)	1·019 (1·004–1·034; p<0·05)	1·017 (1·003–1·030; p<0·05)	0·908 (0·891–0·925; p<0·01)
	Urbanisation rate	1·012 (0·989–1·036; p>0·1)	1·018 (0·993–1·042; p>0·1)	1·007 (0·994–1·021; p>0·1)	1·002 (0·989–1·015; p>0·1)	1·008 (0·996–1·020; p>0·1)	1·022 (0·999–1·047; p<0·1)
	Hospital bed rate per 1000 population	1·150 (1·126–1·174; p<0·01)	1·155 (1·131–1·180; p<0·01)	0·993 (0·984–1·002; p>0·1)	1·002 (0·991–1·013; p>0·1)	1·003 (0·992–1·015; p>0·1)	1·132 (1·109–1·156; p<0·01)
	Rate of physicians per 1000 population	1·022 (1·010–1·034; p<0·01)	1·027 (1·016–1·039; p<0·01)	1·006 (0·992–1·020; p>0·1)	1 (0·984–1·016; p>0·1)	1·002 (0·985–1·020; p>0·1)	1·028 (1·016–1·039; p<0·01)
	Year	Yes	Yes	Yes	Yes	Yes	Yes
Number of observations		73 335	73 371	73 371	73 336	73 372	73 372
Number of municipalities		3669	3671	3671	3669	3671	3671
Avoidable events		8 225 390 (8 192 730–8 257 014)	..	..	713 083 (702 949–723 310)	..	..

Data are RR (95% CI; p value) unless otherwise specified. RR=ratio ratio.

* All covariates are dichotomised based on their mean values, as described in the main manuscript.

Based on these models, we estimated that the number of all-age all-cause deaths avoided during the past two decades (2000–19) due to the implementation of BFP was 713 083 (95% CI 702 949–723 310) and the number of hospitalisations avoided was 8 225 390 (8 192 730–8 257 014) (appendix 2 p 32).

**Figure 1:**
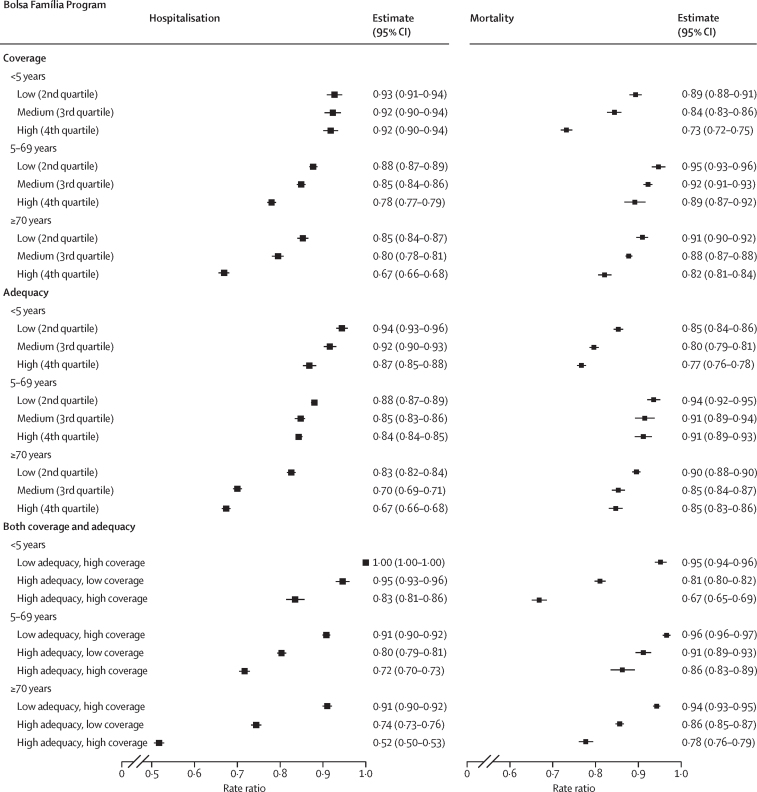
Rate ratios for the association between hospitalisation and mortality rates with Bolsa Família Program coverage and adequacy

Age-stratified models (figure 1) showed reductions in mortality and hospitalisation associated with increasing coverage and adequacy of the BFP in all age groups. The largest observed reductions at the BFP consolidated coverage level were for mortality in children younger than 5 years (hereafter referred to as under-5 mortality), with an RR of 0·73 (95% CI 0·72–0·75), and hospitalisation of people older than 70 years, with an RR of 0·67 (0·66–0·68). Municipalities with high coverage and high adequacy were able to reduce hospitalisation rates of individuals older than 70 years (RR 0·52 [95% CI 0·50–0·53) and under-5 mortality (RR 0·67 [0·65–0·69]). All sensitivity analyses confirmed the robustness of the findings, and all triangulation analyses showed a high degree of confidence in causal inferences (appendix 2 pp 13–31).^25^, ^26^

Projections of expanded BFP eligibility criteria until 2030, as well as three scenarios of BFP coverage—expansion, baseline, and severe fiscal austerity—are explored in appendix 2 (p 34). The forecast for overall mortality rates for the respective austerity scenarios is also presented in appendix 2 (p 34). In the expansion scenario, mortality will decrease over the next decade; in the baseline austerity scenario, rates will slightly increase; and in the severe austerity scenario, mortality rates will significantly increase.

In table 3, RR is reported for the comparison between scenarios. In 2030, the RR between the expansion and baseline scenarios was 0·920 (95% CI 0·919–0·921) for overall mortality and 0·907 (0·907–0·908) for hospitalisations. These RRs corresponded to 683 721 (95% CI 676 494–690 843) averted deaths and 8 046 079 (8 023 306–8 068 416) averted hospitalisations from 2020 to 2030 if expansion strategies were implemented (table 3), instead of keeping their coverage at the baseline level. The RRs and the number of averted deaths that result from modelled combinations of policy responses are comparable in magnitude (appendix 2 pp 33–36).

**Table 3:** Rate ratios and number of avoidable deaths and hospitalisations from the comparison of forecast scenario of expansion versus baseline scenario from 2025 to 2030

	**Hospitalisations**	**Deaths**
**Year**		
2025	0·920 (0·919–0·920)	0·930 (0·929–0·932)
2030	0·907 (0·907–0·908)	0·920 (0·919–0·921)
**Avoidable events**		
2025–30	8 046 079 (8 023 306–8 068 416)	683 721 (676 494–690 843)

Data are rate ratio (95% CI) or n (95% CI).

## Discussion

To the best of our knowledge, this study is the first comprehensive impact evaluation of one of the world's largest CCT programmes on all-age all-cause mortality and hospitalisation, covering its 20-year implementation and integrating projections of the effects of alternative implementation scenarios up to 2030. Our results show that this CCT programme significantly reduced hospitalisations and deaths in Brazil over the past two decades, with the most notable effects observed in under-5 mortality and hospitalisation of individuals older than 70 years. We found that the expansion of BFP has averted 8 225 390 hospitalisations and 713 083 deaths over the last two decades in Brazil and could be able to prevent an additional 8 046 079 hospitalisations and 683 721 deaths up to 2030.

Many studies and reviews have evaluated or summarised the effects of CCT programmes on a wide range of health-related factors, including the use of health services, nutritional status, and health outcomes, often finding positive impacts, although with varying effects depending on the specific programme, country, and contextual characteristics of implementation.^12^, ^13^, ^14^ Furthermore, a 2022 systematic review^11^ found no solid evidence that CCTs are more effective than unconditional cash transfers, suggesting that their effects probably depend on the access to and quality of health-care services provided by the country. Only a few studies have been able to assess the effects of CCTs on mortality. A 2023 global evaluation using data from 37 LMICs associated CCT coverage with a 20% reduction in women's mortality and an 8% decrease in under-5 mortality.^30^ Similarly, evaluations of Mexico's CCT programme, Oportunidades, showed an 11% reduction in maternal mortality (RR 0·890, 95% CI 0·820–0·950) and a 4% decrease in overall mortality.^31^, ^32^ Other studies have indicated reductions in infant mortality linked to CCT programmes in Ecuador and India.^15^, ^16^

In Brazil, previous evaluations have also shown that BFP has been able to reduce child, maternal, and disease-specific mortalities, such as mortality from HIV/AIDS and tuberculosis, especially in populations that are the most vulnerable.^7^, ^14^, ^21^, ^22^ However, to our knowledge, no study has ever comprehensively evaluated the association between the BFP and overall and age-stratified mortality and hospitalisation over the past two decades of implementation. Previous attempts have evaluated the effects of the BFP on overall mortality in shorter periods of time and in specific sub-populations,^8^ or within the framework of the Brazilian welfare state expansion.^2^, ^7^ The success of the BFP in reducing morbidity and mortality in Brazil can be attributed to the multisectoral design of CCT programmes, which integrate direct cash transfers with specific conditionalities. This approach aligns with the Health in All Policies framework,^33^ leading to substantial improvements in population health outcomes and advancing progress towards UN SDG 3 (good health and wellbeing) and its related targets. The BFP can affect overall mortality and morbidity through the income effect and the conditionality effect, that is, by transferring direct income to the beneficiary families, improving families' nutrition and living conditions, and by conditioning the income transfer to the use of basic health services for child and maternal health.^10^ CCTs are also able to improve a wide range of socioeconomic factors that affect health, such as improved education, reduced income inequalities, and social exclusion.^22^ CCT programmes could contribute to psychological and affective pathways that influence health behaviours, for example, by reducing stress and cognitive load, thus enabling more accurate decision making,^34^ particularly regarding food security and health promotion. In municipalities with high programme coverage, the large-scale transfer of resources due to BFP could have important spillover effects on the rest of the community, especially in the poorest regions, substantially improving health outcomes, even for non-beneficiaries of the BFP.^35^

**Figure 2:**
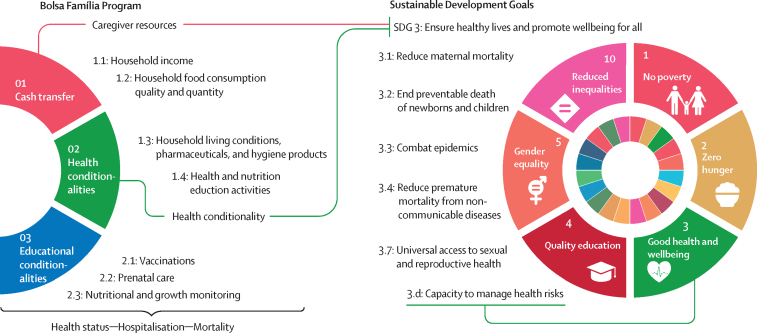
Mechanisms linking the Bolsa Família Program to health outcomes and Sustainable Development Goals

Figure 2 provides a comprehensive description of the mechanisms that could explain the large impact of the BFP on health outcomes (appendix 2 pp 3–5).

The mechanisms that connect the BFP structure to health outcomes align this public policy closely with the UN SDGs, particularly SDG 3: ensure healthy lives and promote wellbeing for all at all ages.^36^ Our results suggest that increased coverage and adequacy of the Brazilian CCT programme are linked to reductions in morbidity and mortality across all age groups, with particularly significant effects on under-5 mortality and hospitalisation of individuals older than 70 years. Regular health monitoring helps prevent diseases and improve the general health of beneficiary families, resulting in improved public health indicators. Monitoring also contributes to regular access for benefiting families to the Unified Health System, affecting overall mortality in the long term. Furthermore, by providing a minimum income to families, BFP also contributes to food security, ensuring that children and adults have access to nutritious food. In the panel, we highlight how this programme aligns with and can contribute to each specific goal.

Panel **Alignment between the Bolsa Família Program (BFP) and the UN Sustainable Development Goal 3 targets**

**Target 3.1: Reduce maternal mortality**

Prenatal care and vaccination monitoring for mothers are some of the health conditionalities of the BFP. Improved financial stability facilitates access to nutritious food and healthier living conditions, reducing risks during pregnancy and childbirth. Previous studies have shown that the risk of maternal death was 18% lower in women who received BFP.^37^

**Target 3.2: End preventable deaths of newborns and children**

Food security provided by BFP through the nutritional monitoring of children younger than 7 years is crucial for the health of newborns and children. Previous studies have shown that increased income allows families to purchase higher quality food and access preventive and curative medical services, decreasing infant mortality.^2^, ^3^, ^38^

**Target 3.3: Combat epidemics**

Several studies have already shown the positive impact of the BFP in reducing the incidence and lethality of AIDS,^39^, ^40^, ^41^ tuberculosis,^42^, ^43^ malaria,^3^, ^44^ and other infectious diseases.^2^, ^3^, ^5^ The programme can fund access to essential treatments and medications for communicable diseases. Improvements in living conditions and basic sanitation resulting from increased income reduces exposure to and spread of neglected tropical diseases and other communicable diseases.^45^, ^46^

**Target 3.4: Reduce premature mortality from non-communicable diseases**

Financial support from BFP can be directed towards preventing and treating non-communicable diseases.^45^, ^46^ The programme facilitates access to healthy food and mental health services, promoting a healthier lifestyle and reducing premature mortality.

**Target 3.7: Universal access to sexual and reproductive health**

Prenatal care is one of the health conditionalities of the BFP, enabling improved pregnancy monitoring.^2^, ^37^, ^47^ Increased family income allows women to have higher access to sexual and reproductive health services, including family planning and education. This increased access results in better reproductive health decisions and integration of these needs into national health programmes.

**Target 3.d: Capacity to manage health risks**

BFP improves the economic conditions of families and facilitates access to health services and information, which can directly strengthen the capacity of people to manage health risks through the adoption of healthier behaviours (eg, healthier food, physical activities, and preventive services) and better living conditions (eg, sanitation, security, and housing). This strengthening, in turn, enhances the national capacity to respond to health emergencies and manage national and global health risks.

The GAAHP of the G20 has increasingly recognised the crucial role of CCT programmes, such as the BFP, in addressing global poverty and inequality, particularly as nations grapple with economic instability, climate change, and post-pandemic recovery. As a forum representing the world's largest economies, the G20 has underscored the importance of innovative financing mechanisms and international cooperation to sustain and expand social protection systems. These efforts include leveraging income transfer programmes to foster inclusive growth, mitigate socioeconomic vulnerabilities, and build resilience in low-income populations. Such global emphasis reaffirms the importance of BFP as a model for integrating poverty alleviation with health and education outcomes, setting a benchmark for achieving the UN SDGs.^2^

This study has limitations. First, the exclusion of municipalities with an inadequate level of vital information could have reduced the external validity of the findings, but it was an essential factor in strengthening its internal validity and reducing biases due to changes in the quality of the death notification system—mainly reduction of sub-notifications—during the study period. However, the municipalities included in the study account for over 87% of Brazil's total population, as those with low-quality vital data are also the least populous. Additionally, these quality criteria are commonly used in similar studies in Brazil and Latin America,^9^, ^11^, ^14^ and our sensitivity analyses showed that the main results were maintained when all municipalities were considered (appendix 2 p 21). Another limitation was the uncertainty of the forecasted scenarios, as the economic and political situation is sometimes volatile. For this reason, different responses to new BFP eligibility criteria were predicted, showing consistent comparison estimates between alternative policy responses.

Another key concern is the possibility of non-random variation in BFP coverage across municipalities, which might be influenced by local implementation capacity and other unmeasured factors related to governance and service provision. To mitigate this, we used municipal fixed-effects regressions to control for time-invariant unobservable characteristics, such as administrative capacity and institutional quality. Furthermore, regressions were controlled by key socioeconomic and policy-related variables to help capture differences in governance, economic conditions, and health-care access, reducing the potential bias associated with local implementation disparities. We also restricted the sample to municipalities with high-quality vital statistics, which minimised biases linked to poor data reporting practices, which are often correlated with weaker governance structures. Additionally, we conducted a robustness check incorporating the Decentralized Management Index for the available years (2015–19), confirming that local implementation differences do not significantly alter our findings. Despite these efforts, residual unobserved variation might persist. Nonetheless, the robustness of our results across multiple sensitivity analyses supports the reliability of our findings.

The main strength of our study was the large range of sensitivity analyses performed, which confirmed the robustness of the findings. The triangulation analyses using difference-in-difference models with propensity-score matching models showed a high degree of confidence in the effect evaluation results^25^ and conferred robustness to the forecasted scenarios of validated models (appendix 2 pp 13–31).

Moreover, our aggregate-level approach, which has been applied in several other impact evaluations of the BFP,^2^, ^3^, ^5^ allowed us to capture the direct effects of CCTs on beneficiaries, as well as the spillover effects on the broader community, which have been shown to be particularly important in the case of the BFP,^48^ and can have substantial additional effects on the municipality's morbidity and mortality rates.^2^, ^3^

In conclusion, our study shows that the expansion of one of the world's largest CCT programme has been able to strongly reduce morbidity and mortality over the past 20 years in Brazil, preventing millions of hospitalisations and deaths. CCT programmes have played a vital role in promoting the health and wellbeing of vulnerable populations in LMICs and have made, and will continue to make, important contributions towards achieving the UN SDG 3 targets by 2030.

### Contributors

### Data sharing

The data used are public and available from the Brazilian Ministry of Health (DATASUS), Brazilian Statistics Institute and Ministry of Social Development websites: http://www2.datasus.gov.br/DATASUS/index.php; https://aplicacoes.mds.gov.br/sagi/vis/data3/data-explorer.php; https://sidra.ibge.gov.br/home/ipca/brasil.

## Declaration of interests

DR and AFS declare funding from the National Institute of Allergy and Infectious Diseases, National Institutes of Health (1R01AI152938). All other authors declare no competing interests.

## Supplementary Materials

Portuguese translation of the summary
