# Supplementary material for: Health effects of the Brazilian Conditional Cash Transfer programme over 20 years and projections to 2030: a retrospective analysis and modelling study
Source: Lancet Public Health. 2025 May 29;10(7):e548–58. doi: 10.1016/S2468-2667(25)00091-X (PMC12208920; doi:10.1016/S2468-2667(25)00091-X)
Supplement: Portuguese translation of the summary [file mmc1.pdf]

THE LANCET
Public Health

Supplementary appendix 1

This translation in Portuguese was submitted by the authors and we reproduce it as supplied. It has not been peer reviewed. *The Lancet*'s editorial processes have only been applied to the original in English, which should serve as reference for this manuscript.

Esta tradução em português foi submetida pelos autores e nós não fizemos quaisquer alterações. Esta versão não foi revista por pares. O processo editorial do The Lancet só foi aplicado à versão original em inglês, que deve servir como referência para este artigo.

Supplement to: Cavalcanti DM, Ordoñez JA, da Silva AF, et al. Health effects of the Brazilian Conditional Cash Transfer programme over 20 years and projections to 2030: a retrospective analysis and modelling study. *Lancet Public Health* 2025; published online May 29. https://doi.org/10.1016/S2468-2667(25)00091-X.

RESUMO

Contexto: Em 2024 o Brasil celebrou o 20º aniversário do Programa Bolsa Família (PBF), um dos mais antigos e maiores programas de Transferência Condicionada de Renda (PTCR) do mundo, abrangendo mais de 50 milhões de brasileiros. Este estudo teve como objetivo avaliar o impacto do PBF nas taxas de mortalidade e hospitalização gerais ao longo das últimas duas décadas, e projetar os efeitos potenciais da expansão deste programa até 2030.

Métodos: Este estudo combinou avaliações retrospectivas de impacto no Brasil de 2000 a 2019 com modelos de microsimulação até 2030. Primeiramente, estimamos o impacto do PBF nas taxas de mortalidade e hospitalização gerais em diferentes faixas-etárias, ajustando para todos os fatores demográficos, socioeconômicos e de saúde relevantes. Utilizamos modelos multivariáveis de *Poisson* com efeitos fixos em 3.671 municípios com qualidade adequada das estatísticas vitais. As três variáveis de exposição do PBF foram (1) cobertura-alvo, (2) adequação dos benefícios (transferência média por família) e (3) interação entre cobertura e adequação. Realizamos várias análises de sensibilidade e triangulação, incluindo modelos de diferença-em-diferença com pareamento por escore de propensão. Em seguida, integramos conjuntos de dados longitudinais anteriores com modelos de microsimulação dinâmica validados para projetar tendências até 2030.

Resultados: Alta cobertura do PBF foram associadas a uma redução estatisticamente significativa nas taxas de mortalidade geral padronizadas por idade de 18% (Taxa de Risco (TR): 0,824; IC 95%: 0,807–0,842) e 15% (TR: 0,849; IC 95%: 0,833–0,866), tendo prevenido 8.225.390 (IC 95%: 8.192.730–8.257.014) hospitalizações e 713.083 (IC 95%: 702.949–723.310) mortes no período de 2000 a 2019. Efeitos mais fortes foram encontrados em municípios com alta cobertura e alta adequação, resultando em uma redução na mortalidade de 33% (TR: 0,669; IC 95%: 0,652–0,687) em crianças menores de cinco anos, e uma redução nas hospitalizações de 48% (TR: 0,517; IC 95%: 0,505–0,529) em pessoas com mais de 70 anos. A expansão da cobertura do PBF poderia evitar 8.046.079 (IC 95%: 8.023.306–8.068.416) hospitalizações e 683.721 (IC 95%: 676.494–690.843) mortes até 2030, em comparação com cenários de cobertura reduzida.

Interpretação: PTCR têm contribuído fortemente para a redução da morbimortalidade no Brasil, prevenindo milhões de hospitalizações e mortes nas últimas duas décadas. Durante o atual período de múltiplas crises, a expansão dos PTCR em termos de cobertura e benefícios poderia evitar muitas hospitalizações e mortes em todo o mundo, devendo ser considerada uma estratégia crucial para alcançar o ODS 3 relacionado à saúde.

Financiamento: *Medical Research Council* (MRC-UKRI), Número da concessão: MC_PC_MR/T023678/1.
