# Supplementary material for: Health effects of the Brazilian Conditional Cash Transfer programme over 20 years and projections to 2030: a retrospective analysis and modelling study
Source: Lancet Public Health. 2025 May 29;10(7):e548–58. doi: 10.1016/S2468-2667(25)00091-X (PMC12208920; doi:10.1016/S2468-2667(25)00091-X)
Supplement: Supplementary appendix 2 [file mmc2.pdf]

THE LANCET
Public Health

Supplementary appendix 2

This appendix formed part of the original submission and has been peer reviewed. We post it as supplied by the authors.

Supplement to: Cavalcanti DM, Ordoñez JA, da Silva AF, et al. Health effects of the Brazilian Conditional Cash Transfer programme over 20 years and projections to 2030: a retrospective analysis and modelling study. *Lancet Public Health* 2025; published online May 29. https://doi.org/10.1016/S2468-2667(25)00091-X.

Supplementary Materials for:

The health impact of one of the world's largest Conditional Cash Transfers in the SDG era: the first 20 years and projections to 2030

TABLE OF CONTENTS

PART I – SUMMARY OF SOCIAL PROGRAMS, DATA SOURCES AND GENERAL METHODOLOGY 2

1. Background – Cash Transference Programs in Brazil 2

1.1. *Bolsa Familia Program (BFP) and Auxílio Brasil Program (ABP)* 2

1.2. Mechanism of the effect of CCT programs on health outcomes 3

2. Dataset 6

2.1. Data sources 6

2.2. Interpolation and extrapolation method 6

2.3. Quality of Vital Statistics Method 9

2.3.1. Discussion and Results – quality of CRVS method 9

PART II – RETROSPECTIVE ANALYSIS 11

3. Empirical methods 11

3.1. Poisson regression – Fixed Effects 11

3.2. Brief Note on the Importance of Using Categorical Variables 11

4. Results 11

4.1. Descriptive Analysis 11

4.2. Retrospective Analysis 13

4.3. Triangulation – Difference-in-difference 13

4.4. Fit and sensitivity tests 14

4.5. Hospitalization and Deaths averted by CCT programs during 2000-2019 32

PART III – FORECASTING ANALYSIS 33

5. Description of the forecasting methodology 33

6. Purpose of the forecasting and its applications 33

7. Inputs, outputs, and other parameters 33

7.1. Scenarios of poverty and coverage of BFP programs 33

8. Prediction methodology 34

9. External validation of the model. 35

10. Sensitivity analysis 35

11. Main Limitations 36

REFERENCES 37

PART I – SUMMARY OF SOCIAL PROGRAMS, DATA SOURCES AND GENERAL METHODOLOGY

1. Background – Cash Transference Programs in Brazil

1.1. Bolsa Familia Program (BFP) and Auxílio Brasil Program (ABP)

The Brazilian CCT program, branded as Bolsa Família Programme (BFP), established in 2004, is an important socio-economic intervention that aims to attenuate the effects of absolute poverty through a minimum cash transfer for beneficiary families, and to break the intergenerational cycle of poverty through investment in education and health conditionalities.[1]

This important socio-economic intervention was established in 2004 by Law № 10,836, of January 9, 2004, with the last monetary restatement modified by Decree № 9,396, of May 30, 2018, in which households are eligible for the program if their per capita income is equal to or less than R$89.00 (approximately US$19 at current 2022 prices) or if they are poor families with income up to R$ 178.00 (approximately US$ 38 at current 2022 prices) and one member is a child up to 17 years old or a pregnant woman (or a woman who just gave birth).[2]

The BFP is one of the largest CCT in the world with more than 13.9 million families benefiting throughout Brazil and was recently remodeled and called Axílio Brasil Program (ABP) by Law № 14,284, of December 29, 2021. Basically, this new program maintains the conditionalities and general structure of the BFP, but it increases the coverage and the value transferred through changes in the criteria of poverty and extreme poverty; where families earning up to R$105.00 per capita (approximately US$22 at current 2022 prices) and up to R$210.00 (almost US$45 at current 2022 prices) are considered extremely poor and poor, respectively.

WEB FIGURE 1 – Number of Beneficiary Families of the Bolsa Família Program (BFP) over the years. Brazil, 2004-2024 (values in millions of families).

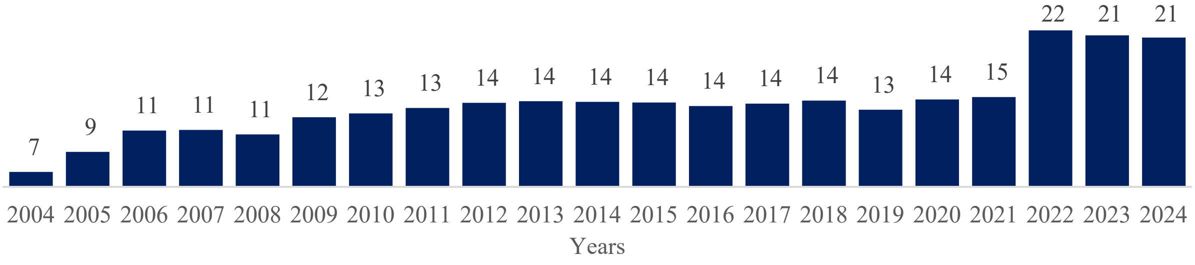

WEB FIGURE 2 – Coverage of BFP. Brazil, 2004-2024.

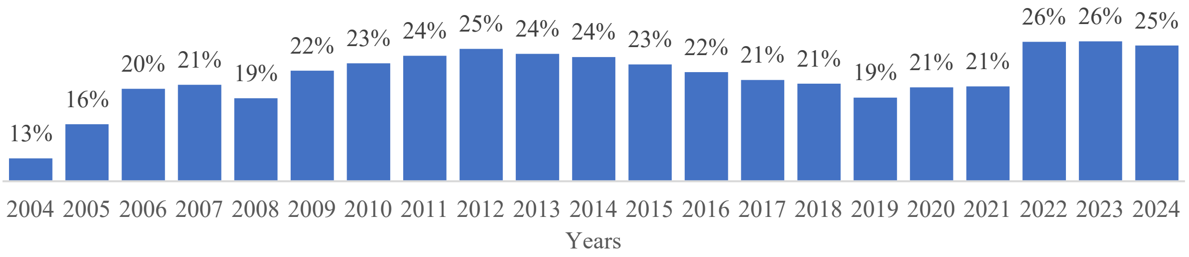

WEB FIGURE 3 – Average amount of money transferred monthly per family (adequacy). BFP, Brazil, 2004-2024.

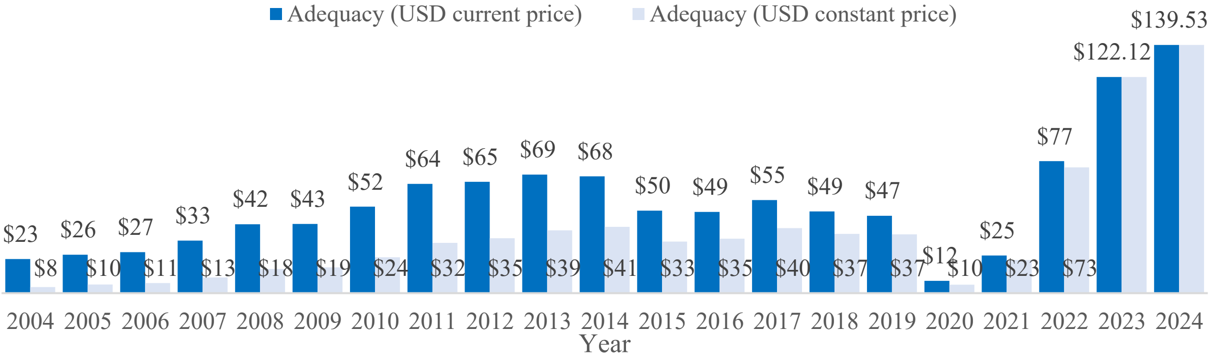

WEB FIGURE 4 – BFP budget over the years. Brazil, 2004 to 2024 (values in USD billions).

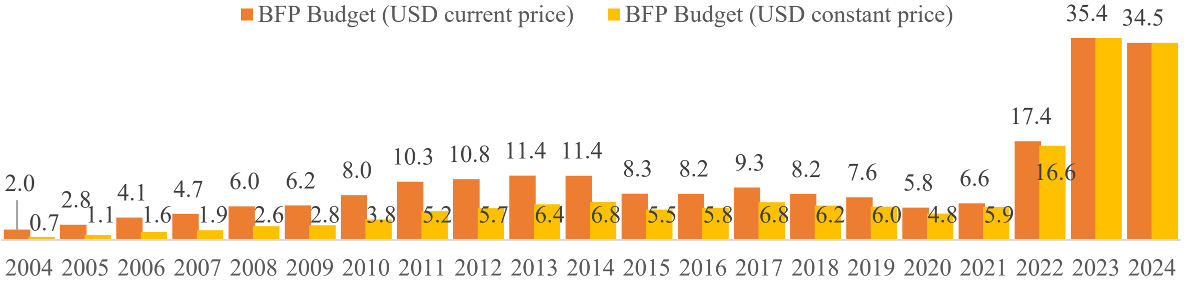

WEB FIGURE 5 – BFP budget as a percentage of Brazilian GDP. Brazil, 2004 to 2024.

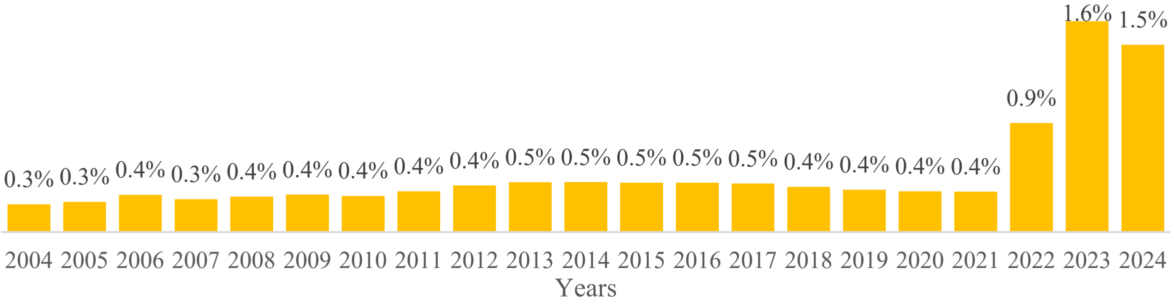

In the health area, conditionalities concern the monitoring of vaccination and nutritional surveillance of children, as well as prenatal care of pregnant women and the puerperium, and should be attended at the Family Health Strategy (*Estratégia de Saúde da Família*, ESF, in Portuguese) units. Some studies have shown the effect of BFP on child morbidity and mortality,[3] and on other health outcomes associated with poverty, such as tuberculosis, leprosy, [1] mortality from suicides, and homicides.[4]

1.2. Mechanism of the effect of CCT programs on health outcomes

There are several mechanisms through which the CCT program affect health outcomes. First, the CCT conditions beneficiaries to a minimum usage of health services for child and maternal health, i.e., the conditionality effect.[3,9] Second, the income transferred to poor and extremely poor families improves the nutrition and living conditions of these families, i.e, the income effect.[1] Third, long-term exposure to the health conditionality of the CCT promote behavior changes and adherence of beneficiary families towards health care.[10]

The results found may point to the PBF's potential to contribute to achieving some of the UN's Sustainable Development Goals (SDG) linked to SDG 3: Health and Well-Being. Regular health monitoring helps prevent diseases and improve the general health of beneficiary families, resulting in better public health indicators. It also contributes to regular access for benefiting families to the

Unified Health System, affecting overall mortality in the long term. Furthermore, by providing a minimum income to families, the PBF also contributes to food security, ensuring that children and adults have access to nutritious food. Below, we highlight how this program can contribute to each specific target:

Target 3.1: Reduce Maternal Mortality. Prenatal care and vaccination monitoring for mothers are some of the BFP's health conditionality. Moreover, greater financial stability facilitates access to nutritious food and healthier living conditions, reducing risks during pregnancy and childbirth. Previous studies show that the risk of maternal death was 18% lower in women who received BFP.21

Target 3.2: End Preventable Deaths of Newborns and Children. The food security provided by BFP through the nutritional monitoring of children under 7 is crucial for the health of newborns and children under 5. Previous studies show that increased income allows families to purchase higher-quality food and access preventive and curative medical services, decreasing infant mortality.2,3,22

Target 3.3: Combat Epidemics. Several studies have already demonstrated the positive impact of the PBF in reducing the incidence and lethality of AIDS,23–25 Tuberculosis,26,27 Malaria,3,28 among others.2,3,5 The program can fund access to essential treatments and medications for communicable diseases, and the improvement in living conditions and basic sanitation resulting from increased income reduces exposure to and spread of neglected tropical diseases and other communicable diseases.29,30

Target 3.4: Reduce Premature Mortality from Non-Communicable Diseases. Financial support from Bolsa Família can be directed towards preventing and treating non-communicable diseases.29,30 Access to healthy food and mental health services is facilitated, promoting a healthier lifestyle and reducing premature mortality.

Target 3.7: Universal Access to Sexual and Reproductive Health. One of the PBF's health conditionality is prenatal care, enabling better pregnancy monitoring. Moreover, increased family income allows women greater access to sexual and reproductive health services, including family planning and education. This results in better reproductive health decisions and greater integration of these needs into national health programs.

Target 3.d: Capacity to Manage Health Risks. BFP improved the economic conditions of families and facilitated access to health services and information, which can directly strengthen the capacity of people to manage health risks through the adoption of healthier behaviours (healthier food, physical activities, preventive services) and better living conditions (sanitation, security, housing). This strengthening, in turn, enhances the country's capacity to respond to health emergencies and manage national and global health risks.

In summary, BFP plays a crucial role in promoting the health and well-being of vulnerable populations in Brazil, significantly contributing to the achievement of the goals set out in SDG 3, and also to other SDGs without a direct link to health such as Gender Equality (SDG 5), Eradicate Poverty (SDG 1), Zero Hunger and Sustainable Agriculture (SDG 2), Reducing Inequalities (SDG 10), among other SDGs of the UN 2030 Agenda.

WEB FIGURE 6. Mechanisms linking the Bolsa Família Program to health outcomes and SDG

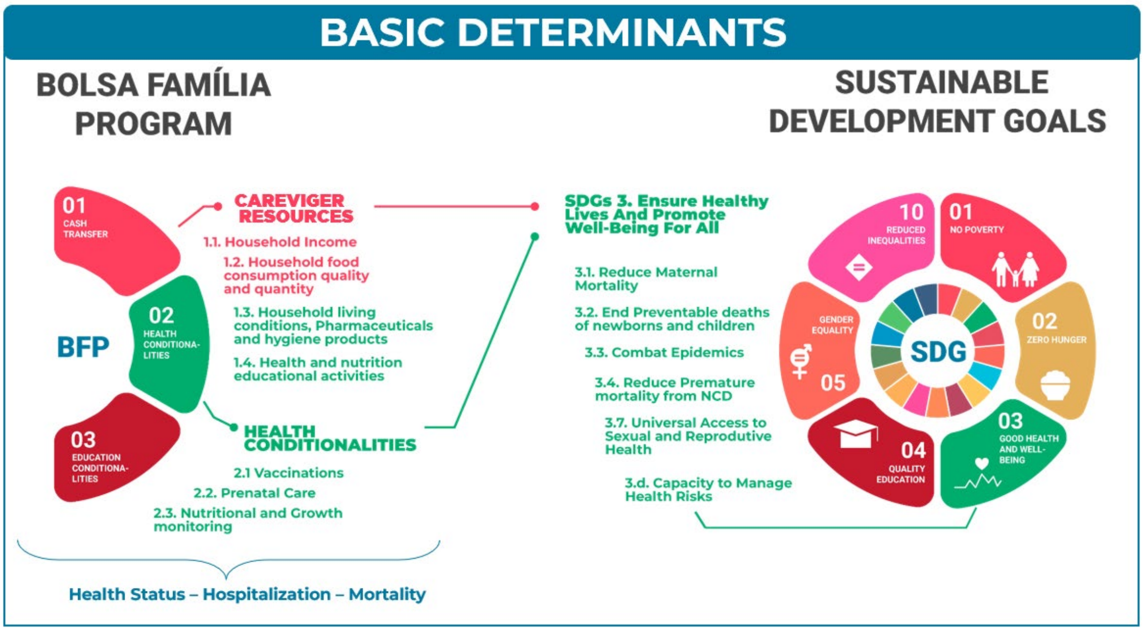

2. Dataset

2.1. Data sources

The data used in this study were obtained from various governmental platforms detailed in Web Table 1. All the variables used in this study are aggregated to the municipal level. However, the data for some variables were not available for specific years and municipalities, therefore we performed a exponential decay method for interpolation, as detailed in Section 2.2. of this supplementary document.

WEB TABLE 1. Data sources and description of variables

VARIABLE	Years	Units of analysis	Source	Link
Mortality and Morbidity	2000 to 2023	Individual	DATASUS - SIM	LINK 1
Population estimates	2000 to 2023	Municipal	IBGE – Census	LINK 2
Livebirth	2000 to 2023	Municipal	DATASUS - SINASC	LINK 3
Socioeconomic variables	2000 and 2010, 2001-2023	State and Municipal	Census; - PNAD; and – PNADC (IBGE)	LINK 4
CCT coverage	2004 to 2023	Municipal	MDS	LINK 5
Hospital bed rate (beds per 1,000 inhabitants)	2000 to 2023	Municipal	DATASUS - CNES	LINK 6
Doctor rate (Physicians per 1,000 inhabitants)	2000 to 2023	Municipal	DATASUS - CNES	LINK 7

Note: DATASUS - Department of Informatics of the Unified Health System (Departamento de Informática do Sistema Único de Saúde); SIM - Mortality Information System (Sistema de Informações sobre Mortalidade); DAB - Department of Primary Care (Departamento de Atenção Básica); CNES - National Register of Health Establishments (Cadastro Nacional de Estabelecimentos de Saúde; SINACS - Live Birth Information System (Sistema de Informações sobre Nascidos Vivos); IBGE - The Brazilian Institute of Geography and Statistics (Instituto Brasileiro de Geografia e Estatística); MDS - The Ministry of Social Development and Fight against Hunger (Ministério do Desenvolvimento Social e Combate à Fome); PNAD - (Pesquisa Nacional por Amostra de Domicílios); PNADC - Continuous PNAD Quarterly (Pesquisa Nacional por Amostra de Domicílios Contínua).

2.2. Interpolation and extrapolation method

Some of control variables are not available in total period of 2000-19. In these cases, we use the exponential decay method to extrapolate the variables available at least two points of time, and we drop municipalities with only one information.

A total of 893,214 values were generated for 7 control variables, in 8,103 municipalities over 20 years with this method. The interpolated variables were Illiteracy rate, household infrastructure (sewage and piped water), hospital bed rate, doctor rate and inequality and income variables (Gini index and poverty rate). Some of these variables were used as control variables for the models, none of the outcome variables (mortality and morbidities) or exposure variables (CCT programs) were interpolated. In the end, it was observed that the interpolated/extrapolated variables improved the control and precision of the retrospective and predictive models.

WEB FIGURE 7. Interpolated and Extrapolated control variables boxplot for selected municipalities in Brazil, from the period 2000-19.

GINI INDEX

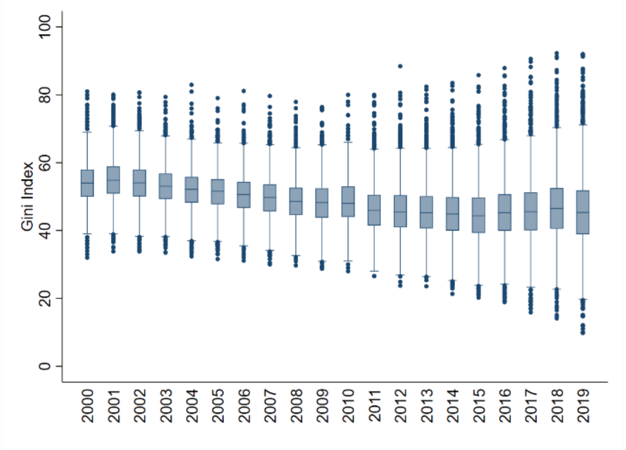

ILLITERACY RATE

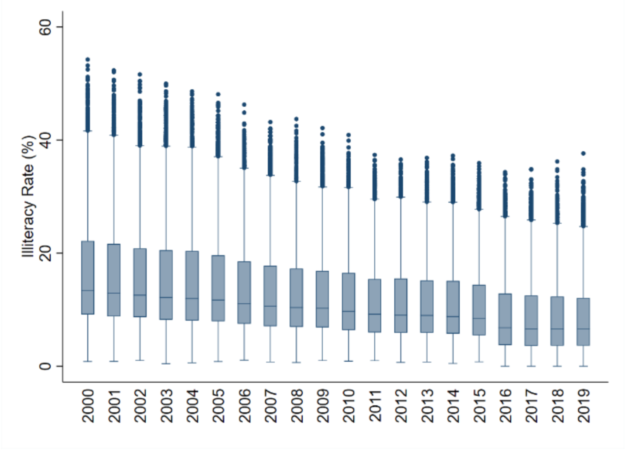

POVERTY RATE

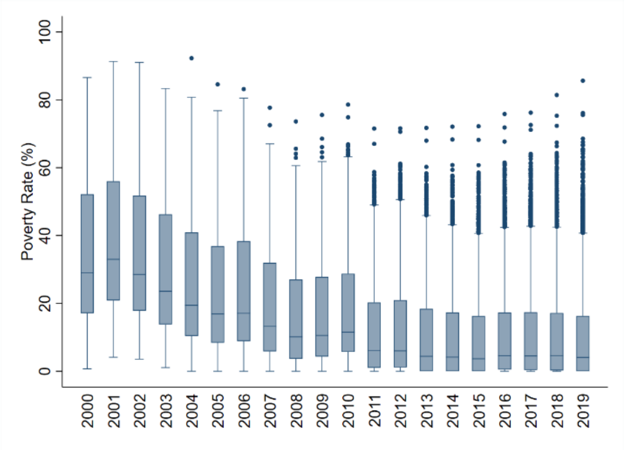

URBANIZATION

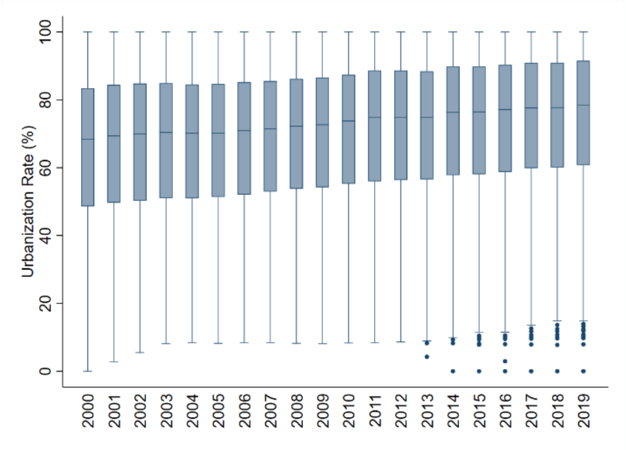

PIPED WATER

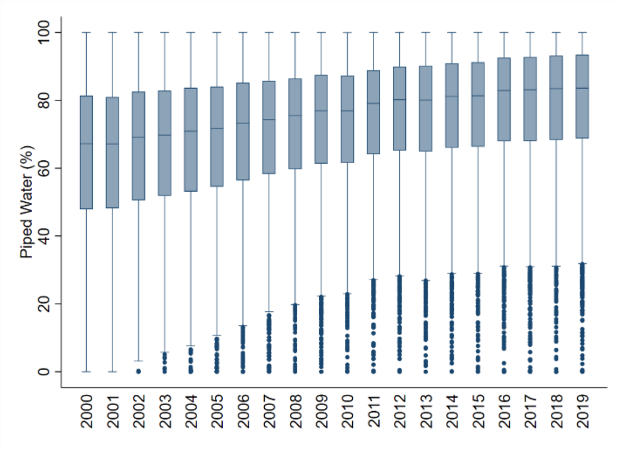

Source: Author's plot for 3,669 selected municipalities in Brazil, over 20 years (from 2000 to 2019).
Note: This variable refers to number of hospital beds per 1,000 inhabitants. We selected municipalities with adequate quality of civil registration and vital statistics (CRVS).

WEB FIGURE 8. Non-Interpolated or Extrapolated control variables boxplot for selected municipalities in Brazil, from the period 2000-19.

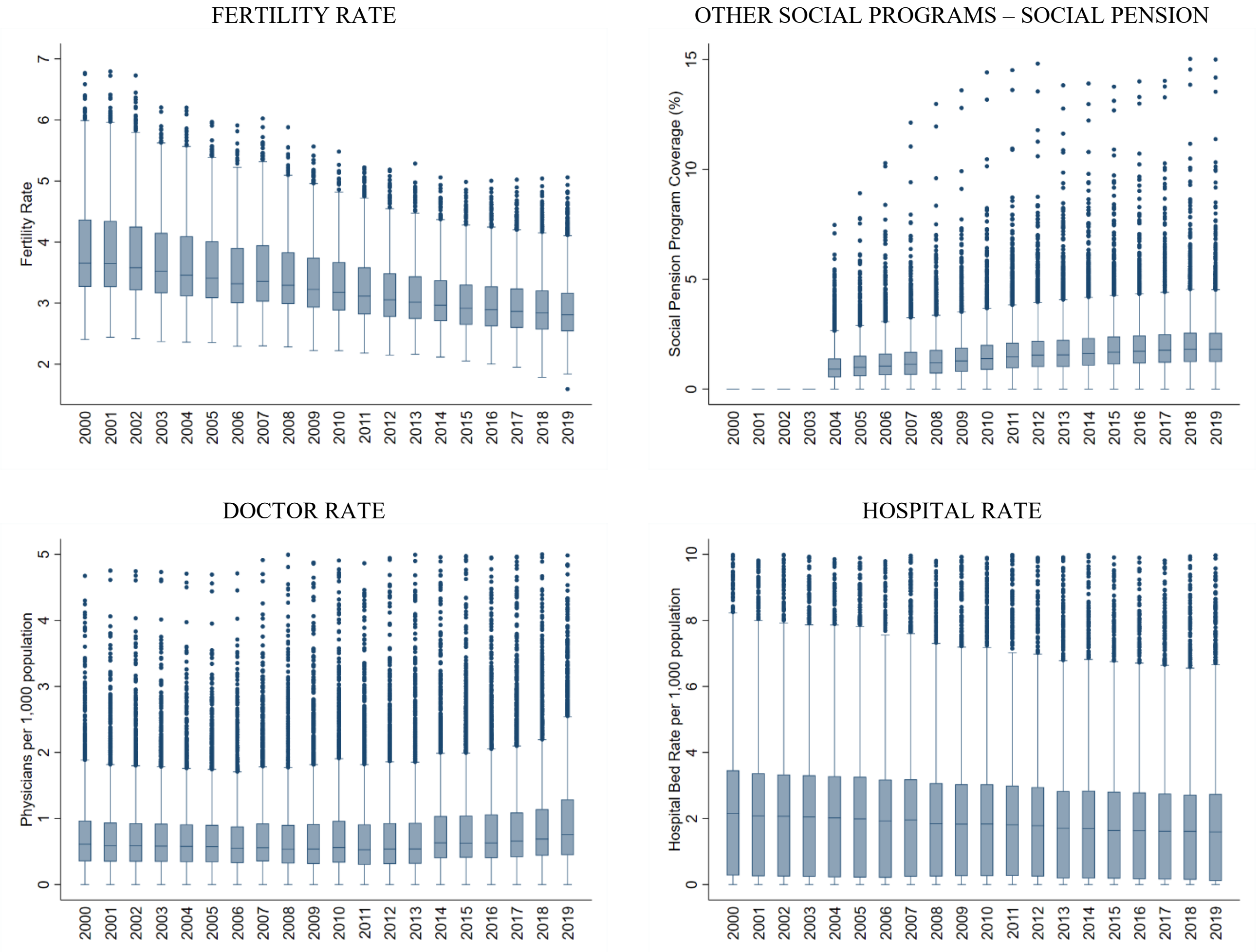

Source: Author's plot for 3,669 selected municipalities in Brazil, over 20 years (from 2000 to 2019).
Note: This variable refers to number of hospital beds per 1,000 inhabitants. We selected municipalities with adequate quality of civil registration and vital statistics (CRVS).

2.3. Quality of Vital Statistics Method

The civil registration and vital statistics (CRVS), despite of being an important public health instrument for planning and evaluation, in most of the developing countries have still low quality and coverage.[14] In that regard, a study[15] involving 148 countries shows that the most of places with medium to low quality of CRVS during the period 1980-2012 were in the African, Asian Latin America regions, which reinforces the necessary care and attention of studies that work with CRVS in these regions. However, countries have been encouraged to stimulated by the need to monitor progress and accountability of CRVS, especially since 2015 for the Sustainable Development Goals (SDG).[14]

From a methodological point of view, the good quality of these CRVS is also crucial to guarantee the veracity of the results, especially in impact evaluation studies. Since our study uses municipal-level child death and livebirth data in LACs countries, it is essential to separate those municipalities with high and low quality from these CRVS.

To mitigate this, we followed a methodology[16] to calculate the level of quality of CRVS of municipalities, which has been widely used in previous studies in Latin America and Caribbean (LAC) countries.[5,17] This methodology used a validated multidimensional criterion based on five indicators: [1] relative mean deviation of the birth-rate; [2] ratio of reported-to-estimated livebirths; [3] age-standardized mortality rate; [4] relative mean deviation of the mortality rate; and [5] proportion of deaths with undetermined causes (Chapter XVIII, ICD-10).[16]

After calculating these indicators, we made a weighted average of them to obtain a final indicator, followed by the division by terciles of the distribution of each country separately, so that the municipalities within the last two terciles were considered to have good quality of CRVS, and the first tercile refer to municipalities with low quality of CRVS.

As the quality CRVS tends to improve in more recent periods, we chose to apply this methodology in the period from 2000 to 2012, as it is the beginning of the historical series worked on in this paper and, at the same time, captures the beginning of the implementation of CCT programs.

2.3.1. Discussion and Results – quality of CRVS method

After applying this method to all 5,507 municipalities in Brazil with data available in 2000-19 period, we selected a subset of 3,669 municipalities that had adequate quality of CRVS, which coverage 67% of them, but represented 87% of the more than 178 million inhabitants in 2002.

Additionally, the application of this method showed important socio-spatial inequalities: In Brazil, the proportion of adequate vital statistics was higher in the Center-South of the country and in the larger municipalities, and lower in many municipalities in the North of the country (Amazon region) and some municipalities in the Northeast. Web-Table 2 and Web-Figure 9 show these results.

WEB TABLE 2. Number of municipalities and population before and after filter by adequate CRVS.

COUNTRY	NUMBER OF MUNICIPALITIES			BY POPULATION		
	WITH DATA AVALIABLE DURIN 2000-19 PERIOD	FILTER BY ADEQUATE QUALITY OF CRVS (2000-02)	%	TOTAL	FILTER BY ADEQUATE QUALITY OF CRVS (2000-02)	%
Brazil	5,507	3,669	67%	178,135,381	155,200,666	87%

Source: Author's analysis of data from 2000-02 from SIM (DATASUS – Brazil).

WEB FIGURE 9. Municipalities according to the quality of vital information

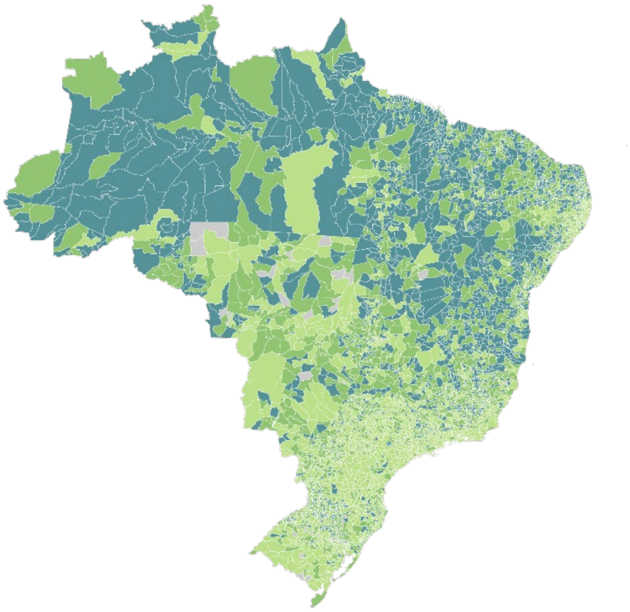

Low quality of CRVS   Good quality of CRVS   High quality of CRVS   Municipalities created after 2001 or other countries not contemplated in this study

Source: Author's analysis of data from 2000-02 from SIM (DATASUS – Brazil).

PART II – RETROSPECTIVE ANALYSIS

3. Empirical methods

3.1. Poisson regression – Fixed Effects

We estimate Fixed Effect models using the Poisson method to retrospectively evaluate and forecast the impact of each welfare social policy on health outcomes. The equation which describes the linear relationship between the health outcomes (mortality and hospitalization rates) and covariates is given by:

$$Log\, Y_{it} = \alpha_i + \sum_{q=1}^{3} \beta_q CCT_{qit} + \sum_{s=4}^{9} \beta_s T_y + \sum_{k=10}^{16} \beta_k X_{kit} + u_{it}$$

where:

- t refers to the year, i refers to an individual municipality, and q are indexes representing each categories of CCT programs coverage.
- Y_{it} are the different welfare state variables (mortality and hospitalization rates for under-five age group) observed at the municipality i in year t.
- CCT_{qit} are the dummies representing the CCT coverage categories (4 groups) observed at the municipality i in year t with a coefficient of β_q; with q=0 for low coverage (0–29.9%), q=1 for intermediate (30–69.9%), q=2 to high (70–99.9%), and q=3 for consolidated coverage (>=100%).
- T_y, are dummy variables representing previous crisis events with coefficients $\beta_4, \beta_9, \beta_{10}$ from the specific years y , with y=2003, 2004, 2008, 2013, 2014, 2015, respectively.
- X_{kit} represents different control covariates, each one with a coefficient of β_k (Poverty, Gini Index, illiteracy, doctors rates, hospital bed rate, Proportion of individuals living in households with inadequate sanitation, and Piped water).
- α_i is the fixed effect (time-invariant) term for each municipality, and u_{it} was the error term.

3.2. Brief Note on the Importance of Using Categorical Variables

In this study, as in the consolidated literature of similar nationwide impact evaluations of public policies using aggregate-level data, [1–10] we preferred to use categorized variables for many reasons. *First*, it provides implementation thresholds more understandable and useful for policy making purposes. *Second*, it reduces the influence of over dispersed values or outliers from the existing or extrapolated data of the independent variables, including main exposures and covariates. *Third*, and most importantly, when the main exposure variable is a categorical dummy variable with multiples levels, it allows to more clearly evaluate the **dose-response** relationship between the exposure and outcome variables, especially if the relationship is non-linear. In this context, the dose-response analysis explores how varying levels of Bolsa Família Program coverage (the "dose") correspond to changes in age-standardized mortality and hospitalization rates (the "response"). This approach, widely applied in epidemiology and public health[1–10] helps determine whether higher program coverage leads to progressively stronger effects, providing additional elements for a causal interpretation of the statistical associations.

4. Results

4.1. Descriptive Analysis

In this section, we present figures (Web Figures 10) that describe the temporal dynamics of the CCT programs from the year 2000 to 2019. We emphasize the difference between CCT crude coverage and CCT target coverage, in which the crude coverage is in relation to the total population of the municipality, while the target is in relation to the eligible poor population of the municipality. We know that the eligibility of each of the CCT programs depends on other factors besides the poverty criterion, however poverty is one of the main criteria. Thus, the term "target" is relative, being more

focused on the population possibly benefited by these CCT programs compared to the total population of the municipality.

WEB FIGURE 10. CCT crude coverage boxplot for selected municipalities in Brazil, Ecuador and Mexico, BEM, from the period 2000-19.

BFP CRUDE COVERAGE

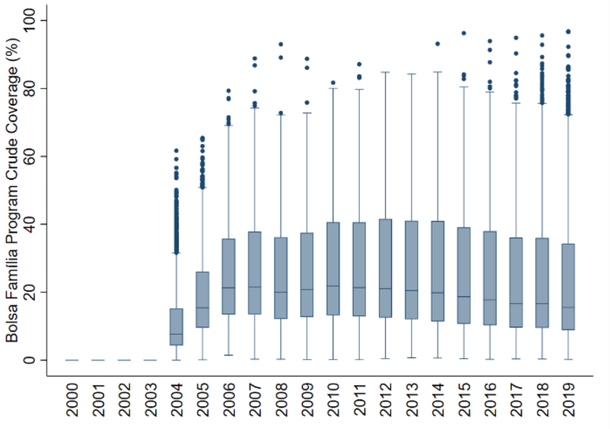

PBF TARGET COVERAGE (current poverty line)

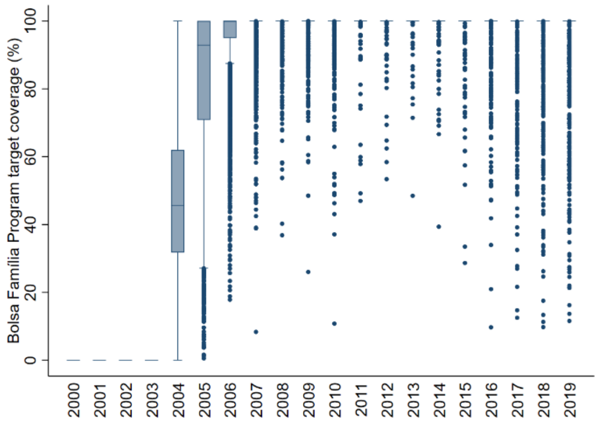

PBF TARGET COVERAGE(New Poverty Line)

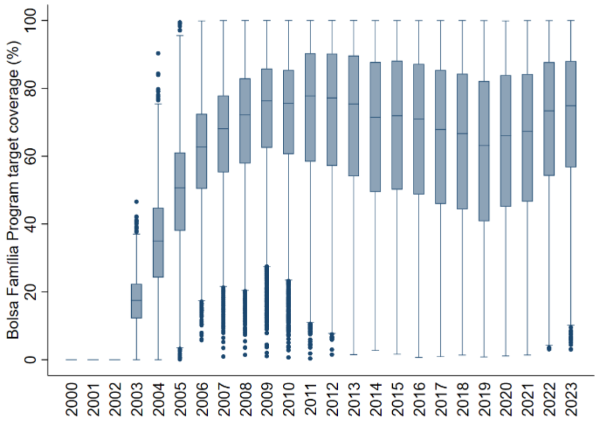

Source: Author's plot for 3,669 selected municipalities in Brazil, over 20 years (from 2000 to 2019).
Note: This variable refers to the conditional cash transference (CCT) programs coverage in relation to the total population of the municipality. We selected municipalities with adequate quality of civil registration and vital statistics (CRVS).

4.2. Retrospective Analysis

4.3. Triangulation – Difference-in-difference

We analysed the effect of BFP on overall mortality and hospitalization rate by difference-in-difference (DID) as a triangulation approach,[18] using the municipalities with no or low CCT coverage since 2004 (n=1,175), versus medium and high coverage municipalities since 2004 (2,216), totaling 3,391 municipalities analyzed in the years 2004 and 2019.

We chose 2004 as the initial year of this analysis because this is the first year of implementation of the *Bolsa Família* Program. The separation between low and high BFP coverage is the same used in the main regressions aggregating the highest categories, that is: low (0–29.9%), intermediate to high and consolidated (30–100%). Thus, municipalities with coverage less than 30% receive a value equal to 0 (control) and municipalities with coverage above 30% receive a value equal to 1 (treated). So that treatment status does not change between treated municipalities, we add a treatment duration effect, i.e., we filter municipalities that have no or low BFP target coverage during the first 4 years (control) and municipalities with medium or high coverage of the BFP in at least 4 years (treated). We tested different duration periods, such as 8 years or more, as well as testing different cutoffs to separate treated municipalities from control municipalities. We follow the step-by-step procedure described in the World Bank handbook, which allows estimating DID with a Poisson panel of fixed effects and with coefficients in rate ratio (RR).

Web-Table 3 shows the result of DID, coverages of BFP programs were associated with a statistically significant reduction in overall hospitalization and mortality rates, with rate ratios (RR) of 0.986 (95%CI: 0.986-0.987) and 0.985(95%CI: 0.984-0.985), respectively. Thus, the BFP programs contributed to the reduction of overall hospitalization and mortality, being compatible with the results already found and described in the main manuscript. Thus, the results by DID with PSM reinforce the results found by the fixed effect panel with Poisson, being a form of triangulation of results.

WEB TABLE 3. Rate Ratios from the difference-in-difference fixed effect Poisson models for the association between overall mortality and hospitalization rates and intermediate to high Conditional Cash Transference (CCT) coverage, in 2004 and 2019.

	Hospitalization	Mortality
**BFP target coverage (dummy)**		
BFP_4_y	1.01346e+12***	2.91400e+13***
	[3.107e+11,3.306e+12]	[1.576e+13,5.390e+13]
Intermediate to high (>=30%) for at least 4 years	0.986***	0.985***
	[0.986,0.987]	[0.984,0.985]
**Control Variables**		
Others social programs	0.981***	0.989***
	[0.978,0.983]	[0.988,0.991]
Poverty rate (%)	0.998***	0.997***
	[0.998,0.998]	[0.997,0.998]
Proportion of individuals older than 15 years who are illiterate (%)	1.002***	0.990***
	[1.001,1.003]	[0.990,0.991]
Gini Index	1.001***	1
	[1.000,1.001]	[1.000,1.000]
Piped water	1.001***	1.001***
	[1.001,1.001]	[1.001,1.001]
Households with inadequate sanitation (%)	0.996***	1.002***
	[0.996,0.997]	[1.002,1.002]
Hospital bed rate per 1,000 population (%)	1.030***	1.003***
	[1.028,1.031]	[1.002,1.004]
Rate of physicians per 1,000 population (%)	1.024***	0.984***
	[1.020,1.028]	[0.982,0.986]
**Number of observations**	69,495	69,496
**Number of municipalities**	3,669	3,669

Source: Author's data analysis for 69,496 observations - 3,669 municipalities in Brazil, over 20 years (from 2000 to 2019).
Note: Data are in Rate Ratio (RR) coefficients (95% CI) unless otherwise specified. The confidence intervals are in parentheses. Time shocks are controls for specific years of economic crisis (2008, 2013, and 2015). The symbols '***' , '**' and '*' denote significance at 1%, 5%, and 10% respectively.

4.4. Fit and sensitivity tests

Concerning our study about the effect of social protection on child mortality, we developed several sensitivity tests to guarantee the robustness of our results.

As a first test for choosing between a random and a fixed effect, we performed **the Hausmann test** for the overall mortality and hospitalization data. By following different goodness of fit criteria including AIC, BIC and the Log-likelihood, we conclude that the fixed effects model was more adequate to analyze the effects of BFP on this kind of mortality and hospitalization. The Web Table 4 shows this test.

Second, we change **the model specification**, measure the pure regression (without covariates), and omit some variables (Web Tables 5, 6 and 19).

Third, to evaluate **the influence of the categorization**, we estimated the models by changing the thresholds by dummy approach (Web Table 7). Shifting the CCT coverage from 4 fixed categories, as defined in the literature, to dummy variables serves to test our models in two key ways: (1st) to determine whether having the exposure variable in four categories while the other covariates are in binary (dummy) form is inflating the results and the program's impact on health outcomes; and (2nd) to assess whether modifying the thresholds of the categorical exposure variable would significantly change the observed outcomes. We also estimated the models using continuous variables (Web Table 8) and tested the behavior of models when switching from CCT target coverage to CCT municipal (crude) coverage, applying both to continuous variables (Web Table 9) and to categorical variables (Web Table 10).

Fourth, to evaluate the **external validity** of our estimates, we fit the models using all 5.507 municipalities (i.e., including municipalities with vital information of lower quality, in Web Table 11).

Fifth, to test **the relevance of the time dummies**, we tested different sets of time variables (Web Table 12 and 13).

Sixth, to evaluate **the stability of the results** with alternative regression models, we fitter Negative Binomial regression models and compare their Akaike Information Criterion (AIC) and Bayesian Information Criterion (BIC) with Poisson models (see Web Table 14). This is reasonable since the Poisson regression takes data overdispersion into account, compared to the Negative Binomial Poisson which does not.

Seventh, we performed a series of heterogeneity of effects analyses, dividing the model by poverty quartiles (see Web Table 15 and 16), and for a larger number of age groups (Web Table 18).

Eight, we estimated a **Falsification/ placebo tests (negative control)**, all aiming to verify unexpected effects on the exposure variable or the dependent variable, using as outcome child mortality due to external causes (ICD10 codes W01-Y99), which CCT are not expected to influence this group of causes and do not include actions for its prevention (see Web Table 17).

Finally, to assess whether PBF coverage was affected by **local implementation capacity** and other unmeasured governance and service delivery factors, we examined the impact of the Decentralized Management Index (IGDM) in the main regressions (see Web-Table 20).In other words, given that our results withstood the vast number of sensitivity tests performed, we conclude that the results and conclusions drawn in this study are robust and stable.

WEB TABLE 4. Hausman test between fixed effect and random effect Poisson models

	Mortality		Hospitalization	
	Fixed Effect	Random effect	Fixed Effect	Random effect
Number of observations	73,336	73,336	73,335	73,335
Number of municipalities	3,669	3,669	3,669	3,669
Log likelihood	-288912.74	-314388.04	-284649.67	-317225.58
Akaike information criterion (AIC)	577859.5	628814.1	569333.3	634489.2
Bayesian information criterion (BIC)	578015.9	628988.9	569489.8	634664
**Hausman test**	**χ^2=1110.16; p-value= 0.000**		**χ^2=1803.80; p-value= 0.000**	

Source: Author's data analysis for 73,336 observations - 3,669 municipalities in Brazil over 20 years (from 2000 to 2019).

WEB TABLE 5. Rate Ratios from the fixed effect Poisson models for the association between overall mortality rates and Conditional Cash Transference (CCT) target coverage program, for Omitted variable bias test, from the period 2000-19, in Brazil.

VARIABLES	Pure regression	Whitou time control	Without selected variables	Without selected variables	All variables
**CCT target population coverage**	1	1	1	1	1
Low (0 – 29.9%)	[1.000,1.000]	[1.000,1.000]	[1.000,1.000]	[1.000,1.000]	[1.000,1.000]
Intermediate (30 – 69.9%)	0.928***	0.924***	0.925***	0.926***	0.924***
	[0.925,0.932]	[0.920,0.928]	[0.921,0.928]	[0.922,0.929]	[0.920,0.927]
High (70 – 99.9%)	0.897***	0.890***	0.889***	0.893***	0.889***
	[0.894,0.901]	[0.886,0.893]	[0.886,0.893]	[0.890,0.897]	[0.885,0.892]
Consolidated (100%)	0.842***	0.827***	0.823***	0.836***	0.824***
	[0.840,0.844]	[0.825,0.829]	[0.821,0.825]	[0.834,0.838]	[0.822,0.826]
**Control Variables**					
Others social programs		0.953***	0.954***	0.951***	0.953***
		[0.949,0.957]	[0.951,0.958]	[0.948,0.955]	[0.950,0.957]
Fertility rate		1.008***	1.006***	0.990***	1.004**
		[1.005,1.012]	[1.002,1.010]	[0.987,0.994]	[1.001,1.008]
Poverty rate (%)		0.964***	0.966***		0.965***
		[0.960,0.968]	[0.962,0.969]		[0.962,0.969]
Proportion of individuals older than 15 years who are illiterate (%)		0.998	0.997	0.986***	0.996*
		[0.993,1.002]	[0.992,1.001]	[0.981,0.990]	[0.992,1.001]
Gini Index		1.023***	1.022***	1.024***	1.020***
		[1.020,1.027]	[1.019,1.025]	[1.021,1.028]	[1.017,1.023]
Piped water		1.018***	1.017***	1.020***	1.019***
		[1.014,1.022]	[1.013,1.020]	[1.016,1.024]	[1.015,1.023]
Households with inadequate sanitation (%)		1.025***	1.025***		1.026***
		[1.021,1.028]	[1.022,1.029]		[1.022,1.029]
Urbanization rate		0.965***		0.964***	0.968***
		[0.959,0.971]		[0.958,0.971]	[0.962,0.974]
Hospital bed rate per 1,000 population (%)		0.994***		0.992***	0.993***
		[0.989,0.998]		[0.988,0.997]	[0.989,0.997]
Rate of physicians per 1,000 population (%)		0.991***		0.992***	0.993***
		[0.988,0.994]		[0.988,0.995]	[0.989,0.996]
**Time trend control**					
2008			1.050***		1.048***
			[1.046,1.053]		[1.044,1.051]
2013					0.998
					[0.994,1.002]
2015					0.979***

VARIABLES	Pure regression	Whitou time control	Without selected variables	Without selected variables	All variables
					[0.975,0.982]
**Number of observations**	73,336	73,336	73,336	73,336	73,336
**Number of municipalities**	3,669	3,669	3,669	3,669	3,669

Source: Author's data analysis for 73,336 observations - 3,669 municipalities in Brazil, over 20 years (from 2000 to 2019).
Note: Data are in Rate Ratio (RR) coefficients (95% CI) unless otherwise specified. The confidence intervals are in parentheses. Time shocks are controls for specific years of economic crisis (2008, 2013, and 2015). The symbols '***' , '**' and '*' denote significance at 1%, 5%, and 10% respectively.

WEB TABLE 6. Rate Ratios from the fixed effect Poisson models for the association between overall hospitalization rates and Conditional Cash Transference (CCT) target coverage program, for Omitted variable bias test, from the period 2000-19, in Brazil.

VARIABLES	Pure regression	Whitou time control	Without selected variables	Without selected variables	All variables
**CCT target population coverage**	1	1	1	1	1
Low (0 – 29.9%)	[1.000,1.000]	[1.000,1.000]	[1.000,1.000]	[1.000,1.000]	[1.000,1.000]
Intermediate (30 – 69.9%)	0.884***	0.887***	0.886***	0.891***	0.885***
	[0.878,0.890]	[0.880,0.893]	[0.879,0.892]	[0.885,0.897]	[0.879,0.892]
High (70 – 99.9%)	0.840***	0.854***	0.850***	0.852***	0.851***
	[0.834,0.846]	[0.848,0.860]	[0.844,0.857]	[0.846,0.858]	[0.845,0.857]
Consolidated (100%)	0.728***	0.768***	0.762***	0.757***	0.768***
	[0.726,0.731]	[0.765,0.772]	[0.759,0.766]	[0.754,0.761]	[0.764,0.771]
**Control Variables**					
Others social programs		0.981***	0.980***	0.984***	0.980***
		[0.977,0.986]	[0.975,0.984]	[0.980,0.989]	[0.976,0.985]
Fertility rate		1.039***	1.038***	1.042***	1.035***
		[1.033,1.045]	[1.032,1.044]	[1.036,1.048]	[1.029,1.041]
Poverty rate (%)		0.965***	0.965***		0.964***
		[0.960,0.971]	[0.960,0.971]		[0.959,0.970]
Proportion of individuals older than 15 years who are illiterate (%)		1.012***	1.011***	1.018***	1.012***
		[1.006,1.019]	[1.005,1.018]	[1.011,1.024]	[1.006,1.019]
Gini Index		1.042***	1.043***	1.037***	1.036***
		[1.037,1.047]	[1.038,1.048]	[1.032,1.042]	[1.031,1.041]
Piped water		0.970***	0.971***	0.960***	0.972***
		[0.965,0.976]	[0.965,0.977]	[0.954,0.966]	[0.967,0.978]
Households with inadequate sanitation (%)		0.880***	0.877***		0.880***
		[0.874,0.885]	[0.872,0.883]		[0.875,0.886]
Urbanization rate		1.001		1.002	1.002
		[0.993,1.009]		[0.993,1.010]	[0.994,1.010]
Hospital bed rate per 1,000 population (%)		1.093***		1.098***	1.091***
		[1.085,1.101]		[1.090,1.106]	[1.083,1.099]
Rate of physicians per 1,000 population (%)		1.011***		1.009***	1.012***
		[1.006,1.016]		[1.005,1.014]	[1.007,1.016]
**Time trend control**					
2008			1.039***		1.031***
			[1.032,1.046]		[1.025,1.038]
2013					0.965***
					[0.958,0.972]
2015					0.950***
					[0.943,0.957]
**Number of observations**	73,335	73,335	73,335	73,335	73,335
**Number of municipalities**	3,669	3,669	3,669	3,669	3,669

Source: Author's data analysis for 73,336 observations - 3,669 municipalities in Brazil, over 20 years (from 2000 to 2019).
Note: Data are in Rate Ratio (RR) coefficients (95% CI) unless otherwise specified. The confidence intervals are in parentheses. Time shocks are controls for specific years of economic crisis (2008, 2013, and 2015). The symbols '***' , '**' and '*' denote significance at 1%, 5%, and 10% respectively.

WEB TABLE 7. Rate Ratios from the fixed effect Poisson models for the association between overall mortality and hospitalization rates with Conditional Cash Transference (CCT) target coverage program categorization changing from fixed category to dummies, from the period 2000-19, in Brazil,.

VARIABLES	Mortality		Hospitalization	
	Pure regression	All variables	Pure regression	All variables
**CCT target population coverage**				
Below median	1	1	1	1
	[1.000,1.000]	[1.000,1.000]	[1.000,1.000]	[1.000,1.000]
Median and above	0.940***	0.975***	0.804***	0.896***
	[0.937,0.942]	[0.972,0.978]	[0.801,0.807]	[0.892,0.900]
**Control Variables**				
Others social programs		0.963***		1.016***
		[0.959,0.968]		[1.011,1.021]
Fertility rate		1.041***		1.081***
		[1.036,1.046]		[1.075,1.088]
Poverty rate (%)		1.040***		1.014***
		[1.035,1.044]		[1.008,1.020]
Proportion of individuals older than 15 years who are illiterate (%)		1.021***		1.037***
		[1.016,1.027]		[1.030,1.044]
Gini Index		1.063***		1.103***
		[1.059,1.067]		[1.098,1.109]
Piped water		0.989***		0.935***
		[0.984,0.993]		[0.929,0.941]
Households with inadequate sanitation (%)		1.003		0.856***
		[0.999,1.007]		[0.851,0.861]
Urbanization rate		0.919***		0.984***
		[0.912,0.926]		[0.976,0.992]
Hospital bed rate per 1,000 population (%)		0.994**		1.105***
		[0.989,0.999]		[1.097,1.114]
Rate of physicians per 1,000 population (%)		0.974***		1.011***
		[0.970,0.978]		[1.006,1.015]
Year binaries	No	Yes	No	Yes
**Number of observations**	73,372	73,372	73,371	73,371
**Number of municipalities**	3,669	3,669	3,669	3,669

Source: Author's data analysis for 73,336 observations - 3,669 municipalities in Brazil, over 20 years (from 2000 to 2019).
Note: Data are in Rate Ratio (RR) coefficients (95% CI) unless otherwise specified. The confidence intervals are in parentheses. Time shocks are controls for specific years of economic crisis (2008, 2013, and 2015). The symbols '***' , '**' and '*' denote significance at 1%, 5%, and 10% respectively.

WEB TABLE 8. Rate Ratios from the fixed effect Poisson models for the association between overall mortality and hospitalization rates with Conditional Cash Transference (CCT) target coverage using continuous variables; from the period 2000-19 in Brazil.

VARIABLES	Mortality		Hospitalization	
	**Pure regression**	**All variables**	**Pure regression**	**All variables**
**CCT target population coverage**	0.997***<br>[0.996,0.997]	0.999***<br>[0.998,0.999]	0.992***<br>[0.992,0.992]	0.997***<br>[0.997,0.998]
**Control Variables**				
Others social programs		0.996***<br>[0.994,0.997]		0.993***<br>[0.991,0.996]
Fertility rate		0.909***<br>[0.902,0.916]		1.057***<br>[1.045,1.069]
Poverty rate (%)		0.998***<br>[0.998,0.999]		0.997***<br>[0.997,0.998]
Proportion of individuals older than 15 years who are illiterate (%)		0.987***<br>[0.987,0.988]		0.994***<br>[0.993,0.995]
Gini Index		1.000**<br>[1.000,1.001]		1.001***<br>[1.000,1.001]
Piped water		1.001***<br>[1.001,1.001]		1.001***<br>[1.001,1.001]
Households with inadequate sanitation (%)		1.002***<br>[1.002,1.002]		0.998***<br>[0.997,0.998]
Urbanization rate		0.999***<br>[0.999,0.999]		1.004***<br>[1.004,1.005]
Hospital bed rate per 1,000 population (%)		1<br>[0.999,1.001]		1.027***<br>[1.026,1.029]
Rate of physicians per 1,000 population (%)		0.996***<br>[0.994,0.998]		1.033***<br>[1.030,1.037]
Year		0.977***<br>[0.976,0.977]		0.977***<br>[0.976,0.977]
**Number of observations**	73,372	73,372	73,371	73,371
**Number of municipalities**	3,669	3,669	3,669	3,669

Source: Author's data analysis for 73,336 observations - 3,669 municipalities in Brazil, over 20 years (from 2000 to 2019).
Note: Data are in Rate Ratio (RR) coefficients (95% CI) unless otherwise specified. The confidence intervals are in parentheses. Time shocks are controls for specific years of economic crisis (2008, 2013, and 2015). The symbols '***' , '**' and '*' denote significance at 1%, 5%, and 10% respectively.

WEB TABLE 9. Rate Ratios from the fixed effect Poisson models for the association between overall mortality and hospitalization rates with Conditional Cash Transference (CCT) crude (municipal) coverage using continuous variables; from the period 2000-19 in Brazil.

VARIABLES	Mortality		Hospitalization	
	**Pure regression**	**All variables**	**Pure regression**	**All variables**
**CCT crude population coverage**	0.998***	0.999***	0.993***	0.997***
	[0.998,0.999]	[0.999,0.999]	[0.993,0.993]	[0.997,0.997]
**Control Variables**				
Others social programs		1.013***		0.998
		[1.010,1.015]		[0.994,1.002]
Fertility rate		0.912***		1.043***
		[0.905,0.920]		[1.029,1.057]
Poverty rate (%)		0.998***		0.996***
		[0.998,0.998]		[0.996,0.997]
Proportion of individuals older than 15 years who are illiterate (%)		0.992***		0.999*
		[0.991,0.993]		[0.998,1.000]
Gini Index		1.001***		1.001***
		[1.000,1.001]		[1.001,1.002]
Piped water		1.001***		1.002***
		[1.001,1.001]		[1.001,1.002]
Households with inadequate sanitation (%)		1.001***		0.995***
		[1.001,1.002]		[0.995,0.996]
Urbanization rate		0.999***		1.005***
		[0.999,1.000]		[1.005,1.006]
Hospital bed rate per 1,000 population (%)		1		1.025***
		[0.999,1.001]		[1.024,1.027]
Rate of physicians per 1,000 population (%)		0.988***		1.024***
		[0.986,0.990]		[1.020,1.028]
Year		0.980***		0.982***
		[0.979,0.980]		[0.982,0.983]
Year binaries	No	Yes	No	Yes
**Number of observations**	58,698	58,698	58,697	58,697
**Number of municipalities**	3,669	3,669	3,669	3,669

Source: Author's data analysis for 73,336 observations - 3,669 municipalities in Brazil, over 20 years (from 2000 to 2019).
Note: Data are in Rate Ratio (RR) coefficients (95% CI) unless otherwise specified. The confidence intervals are in parentheses. Time shocks are controls for specific years of economic crisis (2008, 2013, and 2015). The symbols '***' , '**' and '*' denote significance at 1%, 5%, and 10% respectively.

WEB TABLE 10. Rate Ratios from the fixed effect Poisson models for the association between overall mortality and hospitalization rates with Conditional Cash Transference (CCT) crude (municipal) coverage using categorical variables; from the period 2000-19 in Brazil.

VARIABLES	Mortality		Hospitalization	
	**Pure regression**	**All variables**	**Pure regression**	**All variables**
**CCT target population coverage**	1	1	1	1
First quartile	[1.000,1.000]	[1.000,1.000]	[1.000,1.000]	[1.000,1.000]
Second quartile	0.978***	0.992**	0.954***	0.972***
	[0.975,0.981]	[0.985,0.999]	[0.949,0.960]	[0.967,0.978]
Third quartile	0.976***	0.984***	0.899***	0.934***
	[0.972,0.980]	[0.980,0.988]	[0.893,0.906]	[0.927,0.940]
Fourth quartile	0.977***	0.980***	0.788***	0.861***
	[0.970,0.983]	[0.977,0.983]	[0.780,0.796]	[0.853,0.870]
**Control Variables**				
Others social programs		0.984***		1.009***
		[0.980,0.988]		[1.004,1.015]
Fertility rate		1.006**		1.043***
		[1.001,1.010]		[1.036,1.049]
Poverty rate (%)		0.990***		0.998
		[0.985,0.995]		[0.991,1.006]
Proportion of individuals older than 15 years who are illiterate (%)		1.024***		1.044***
		[1.019,1.030]		[1.036,1.052]
Gini Index		1.033***		1.063***
		[1.030,1.037]		[1.058,1.068]
Piped water		0.993***		0.961***
		[0.988,0.997]		[0.955,0.968]
Households with inadequate sanitation (%)		0.977***		0.860***
		[0.973,0.981]		[0.854,0.866]
Urbanization rate		0.943***		0.977***
		[0.936,0.950]		[0.968,0.986]
Hospital bed rate per 1,000 population (%)		1.003		1.080***
		[0.998,1.008]		[1.071,1.088]
Rate of physicians per 1,000 population (%)		0.980***		1.011***
		[0.976,0.984]		[1.006,1.016]
Year binaries	No	Yes	No	Yes
**Number of observations**	58,698	58,698	58,698	58,698
**Number of municipalities**	3,669	3,669	3,669	3,669

Source: Author's data analysis for 73,336 observations - 3,669 municipalities in Brazil, over 20 years (from 2000 to 2019).
Note: Data are in Rate Ratio (RR) coefficients (95% CI) unless otherwise specified. The confidence intervals are in parentheses. Time shocks are controls for specific years of economic crisis (2008, 2013, and 2015). The symbols '***' , '**' and '*' denote significance at 1%, 5%, and 10% respectively.

WEB TABLE 11. Rate Ratios from the fixed effect Poisson models for the association between overall mortality and hospitalization rates with Conditional Cash Transference (CCT) target coverage using categorical variables, and without filter for adequate information (CRVS); from the period 2000-19 in all municipalities in Brazil.

VARIABLES	MORTALITY		HOSPITALIZATION	
	**Pure regression**	**All variables**	**Pure regression**	**All variables**
**CCT target population coverage**	1	1	1	1
Low (0 – 29.9%)	[1.000,1.000]	[1.000,1.000]	[1.000,1.000]	[1.000,1.000]
Intermediate (30 – 69.9%)	0.941***	0.936***	0.894***	0.899***
	[0.937,0.945]	[0.932,0.940]	[0.889,0.900]	[0.893,0.904]
High (70 – 99.9%)	0.919***	0.904***	0.850***	0.862***
	[0.915,0.922]	[0.901,0.908]	[0.844,0.855]	[0.857,0.868]
Consolidated (100%)	0.878***	0.847***	0.745***	0.781***
	[0.876,0.879]	[0.845,0.849]	[0.743,0.747]	[0.778,0.784]
**Control Variables**				
Others social programs		0.921***		0.970***
		[0.918,0.925]		[0.966,0.975]
Fertility rate		0.966***		1.044***
		[0.962,0.970]		[1.038,1.050]
Poverty rate (%)		0.998		0.975***
		[0.994,1.001]		[0.970,0.980]
Proportion of individuals older than 15 years who are illiterate (%)		0.965***		0.996
		[0.960,0.970]		[0.990,1.002]
Gini Index		1.008***		1.028***
		[1.006,1.011]		[1.024,1.033]
Piped water		1.054***		0.991***
		[1.049,1.058]		[0.985,0.996]
Households with inadequate sanitation (%)		1.020***		0.877***
		[1.017,1.023]		[0.872,0.882]
Urbanization rate		1.011***		1.021***
		[1.004,1.017]		[1.014,1.029]
Hospital bed rate per 1,000 population (%)		1		1.095***
		[0.996,1.003]		[1.089,1.101]
Rate of physicians per 1,000 population (%)		1.004**		1.019***
		[1.001,1.007]		[1.015,1.024]
Year binaries	No	Yes	No	Yes
**Number of observations**	110,080	110,080	110,079	110,079
**Number of municipalities**	5,507	5,507	5,507	5,507

Source: Author's data analysis for 110,080 observations – 5,507 municipalities in Brazil, over 20 years (from 2000 to 2019).
Note: Data are in Rate Ratio (RR) coefficients (95% CI) unless otherwise specified. The confidence intervals are in parentheses. Time shocks are controls for specific years of economic crisis (2008, 2013, and 2015). The symbols '***' , '**' and '*' denote significance at 1%, 5%, and 10% respectively.

WEB TABLE 12. Rate Ratios from the fixed effect Poisson models for the association between overall mortality rates and Conditional Cash Transference (CCT) target coverage using categorical variables, with different time shocks control; from the period 2000-19 in Brazil.

VARIABLES	OVERALL MORTALITY RATE BY DIFFERENT TIME SHOCK CONTROL				
	**No time Control**	**First Year** 2004	**First 5 years** 2005 to 2009	**Second 5 years** 2010 to 2014	**Last 5 years** 2015 to 2019
**CCT target population coverage**	1	1	1	1	1
Low (0 – 29.9%)	[1.000,1.000]	[1.000,1.000]	[1.000,1.000]	[1.000,1.000]	[1.000,1.000]
Intermediate (30 – 69.9%)	0.924***	0.895***	0.904***	0.924***	0.925***
	[0.920,0.928]	[0.890,0.900]	[0.900,0.907]	[0.920,0.928]	[0.922,0.929]
High (70 – 99.9%)	0.890***	0.884***	0.840***	0.888***	0.891***
	[0.886,0.893]	[0.880,0.888]	[0.837,0.844]	[0.884,0.891]	[0.888,0.895]
Consolidated (100%)	0.827***	0.829***	0.800***	0.824***	0.842***
	[0.825,0.829]	[0.827,0.831]	[0.798,0.802]	[0.822,0.826]	[0.840,0.844]
**Control Variables**					
Others social programs	0.953***	0.953***	0.955***	0.953***	0.951***
	[0.949,0.957]	[0.950,0.957]	[0.951,0.958]	[0.950,0.957]	[0.947,0.954]
Fertility rate	1.008***	1.008***	0.994***	1.007***	0.988***
	[1.005,1.012]	[1.005,1.012]	[0.990,0.997]	[1.003,1.011]	[0.984,0.991]
Poverty rate (%)	0.964***	0.966***	0.964***	0.964***	0.971***
	[0.960,0.968]	[0.962,0.969]	[0.960,0.967]	[0.960,0.967]	[0.967,0.974]
Proportion of individuals older than 15 years who are illiterate (%)	0.998	0.997	0.988***	0.997	0.976***
	[0.993,1.002]	[0.992,1.001]	[0.984,0.992]	[0.993,1.002]	[0.972,0.980]
Gini Index	1.023***	1.023***	1.006***	1.023***	1.016***
	[1.020,1.027]	[1.020,1.026]	[1.003,1.009]	[1.020,1.026]	[1.013,1.019]
Piped water	1.018***	1.019***	1.027***	1.019***	1.028***
	[1.014,1.022]	[1.015,1.023]	[1.023,1.031]	[1.015,1.023]	[1.024,1.032]
Households with inadequate sanitation (%)	1.025***	1.025***	1.042***	1.024***	1.045***
	[1.021,1.028]	[1.021,1.028]	[1.038,1.045]	[1.021,1.028]	[1.041,1.048]
Urbanization rate	0.965***	0.966***	0.984***	0.966***	0.991***
	[0.959,0.971]	[0.959,0.972]	[0.978,0.990]	[0.960,0.972]	[0.985,0.997]
Hospital bed rate per 1,000 population (%)	0.994***	0.994***	0.989***	0.993***	0.987***
	[0.989,0.998]	[0.990,0.998]	[0.985,0.993]	[0.988,0.997]	[0.983,0.991]
Rate of physicians per 1,000 population (%)	0.991***	0.991***	1.001	0.992***	1.007***
	[0.988,0.994]	[0.988,0.995]	[0.998,1.005]	[0.989,0.996]	[1.004,1.011]
**Time Control**					
2004		1.049***			
		[1.044,1.054]			
2005			1.072***		
			[1.068,1.076]		
2006			1.090***		
			[1.086,1.094]		
2007			1.082***		
			[1.078,1.086]		
2008			1.071***		
			[1.067,1.075]		
2009			1.055***		
			[1.052,1.059]		
2010				1.025***	
				[1.021,1.029]	
2011				1.029***	
				[1.025,1.032]	
2012				1.007***	
				[1.003,1.011]	
2013				1	
				[0.996,1.003]	
2014				0.984***	
				[0.981,0.988]	

VARIABLES	OVERALL MORTALITY RATE BY DIFFERENT TIME SHOCK CONTROL				
	**No time Control**	**First Year** 2004	**First 5 years** 2005 to 2009	**Second 5 years** 2010 to 2014	**Last 5 years** 2015 to 2019
2015					0.943***
					[0.940,0.946]
2016					0.943***
					[0.940,0.946]
2017					0.915***
					[0.912,0.918]
2018					0.891***
					[0.888,0.894]
2019					0.884***
					[0.881,0.887]
**Number of observations**	73,336	73,336	73,336	73,336	73,336
**Number of municipalities**	3,669	3,669	3,669	3,669	3,669

Source: Author's data analysis for 73,336 observations - 3,669 municipalities in Brazil, over 20 years (from 2000 to 2019).
Note: Data are in Rate Ratio (RR) coefficients (95% CI) unless otherwise specified. The confidence intervals are in parentheses. Time shocks are controls for specific years of economic crisis (2008, 2013, and 2015). The symbols '***' , '**' and '*' denote significance at 1%, 5%, and 10% respectively.

WEB TABLE 13. Rate Ratios from the fixed effect Poisson models for the association between overall hospitalization rates and Conditional Cash Transference (CCT) target coverage using categorical variables, with different time shocks control; from the period 2000-19 in Brazil.

VARIABLES	OVERALL HOSPITALIZATION RATE BY DIFFERENT TIME SHOCK CONTROL				
	**No time Control**	**First Year** 2004	**First 5 years** 2005 to 2009	**Second 5 years** 2010 to 2014	**Last 5 years** 2015 to 2019
**CCT target population coverage**	1	1	1	1	1
Low (0 – 29.9%)	[1.000,1.000]	[1.000,1.000]	[1.000,1.000]	[1.000,1.000]	[1.000,1.000]
Intermediate (30 – 69.9%)	0.887***	0.880***	0.849***	0.886***	0.885***
	[0.880,0.893]	[0.872,0.888]	[0.843,0.855]	[0.879,0.892]	[0.879,0.891]
High (70 – 99.9%)	0.854***	0.853***	0.768***	0.852***	0.852***
	[0.848,0.860]	[0.847,0.859]	[0.762,0.775]	[0.846,0.858]	[0.846,0.858]
Consolidated (100%)	0.768***	0.769***	0.719***	0.768***	0.774***
	[0.765,0.772]	[0.765,0.772]	[0.715,0.722]	[0.764,0.772]	[0.770,0.777]
**Control Variables**					
Others social programs	0.981***	0.981***	0.973***	0.982***	0.970***
	[0.977,0.986]	[0.977,0.986]	[0.968,0.977]	[0.977,0.986]	[0.965,0.974]
Fertility rate	1.039***	1.039***	1.014***	1.037***	1.009***
	[1.033,1.045]	[1.033,1.045]	[1.008,1.019]	[1.032,1.043]	[1.004,1.015]
Poverty rate (%)	0.965***	0.966***	0.962***	0.963***	0.969***
	[0.960,0.971]	[0.960,0.971]	[0.957,0.968]	[0.958,0.969]	[0.964,0.975]
Proportion of individuals older than 15 years who are illiterate (%	1.012***	1.012***	0.998	1.013***	0.993**
	[1.006,1.019]	[1.005,1.019]	[0.992,1.005]	[1.007,1.020]	[0.987,0.999]
Gini Index	1.042***	1.042***	1.011***	1.036***	1.026***
	[1.037,1.047]	[1.037,1.047]	[1.006,1.016]	[1.031,1.041]	[1.021,1.031]
Piped water	0.970***	0.971***	0.988***	0.973***	0.987***
	[0.965,0.976]	[0.965,0.976]	[0.982,0.994]	[0.967,0.979]	[0.981,0.993]
Households with inadequate sanitation (%)	0.880***	0.880***	0.902***	0.879***	0.895***
	[0.874,0.885]	[0.875,0.885]	[0.897,0.908]	[0.874,0.885]	[0.890,0.901]
Urbanization rate	1.001	1.001	1.018***	1.001	1.019***
	[0.993,1.009]	[0.993,1.009]	[1.010,1.026]	[0.993,1.009]	[1.011,1.027]
Hospital bed rate per 1,000 population (%)	1.093***	1.093***	1.080***	1.092***	1.076***
	[1.085,1.101]	[1.085,1.101]	[1.072,1.088]	[1.084,1.100]	[1.068,1.084]
Rate of physicians per 1,000 population (%)	1.011***	1.011***	1.018***	1.011***	1.025***
	[1.006,1.016]	[1.006,1.016]	[1.014,1.023]	[1.006,1.016]	[1.020,1.030]
**Time Control**					

VARIABLES	OVERALL HOSPITALIZATION RATE BY DIFFERENT TIME SHOCK CONTROL				
	**No time Control**	**First Year** 2004	**First 5 years** 2005 to 2009	**Second 5 years** 2010 to 2014	**Last 5 years** 2015 to 2019
y2004		1.012***			
		[1.004,1.020]			
y2005			1.128***		
			[1.120,1.136]		
y2006			1.141***		
			[1.133,1.149]		
y2007			1.142***		
			[1.135,1.150]		
y2008			1.080***		
			[1.073,1.088]		
y2009			1.090***		
			[1.083,1.097]		
y2010				1.038***	
				[1.031,1.045]	
y2011				1.012***	
				[1.005,1.019]	
y2012				0.981***	
				[0.974,0.988]	
y2013				0.966***	
				[0.959,0.973]	
y2014				0.966***	
				[0.959,0.972]	
y2015					0.912***
					[0.906,0.918]
y2016					0.890***
					[0.884,0.896]
y2017					0.888***
					[0.882,0.894]
y2018					0.897***
					[0.890,0.903]
y2019					0.890***
					[0.884,0.897]
**Number of observations**	73,336	73,336	73,336	73,336	73,336
**Number of municipalities**	3,669	3,669	3,669	3,669	3,669

Source: Author's data analysis for 73,336 observations - 3,669 municipalities in Brazil, over 20 years (from 2000 to 2019).
Note: Data are in Rate Ratio (RR) coefficients (95% CI) unless otherwise specified. The confidence intervals are in parentheses. Time shocks are controls for specific years of economic crisis (2008, 2013, and 2015). The symbols '***' , '**' and '*' denote significance at 1%, 5%, and 10% respectively.

WEB TABLE 14. Rate Ratios for the association between overall mortality and hospitalization rates with Conditional Cash Transference (CCT) target coverage, using Negative Binomial models; from the period 2000-19 in all municipalities in Brazil.

VARIABLES	Mortality		Hospitalization	
	**Pure regression**	**All variables**	**Pure regression**	**All variables**
**CCT target population coverage**	1	1	1	1
Low (0 – 29.9%)	[1.000,1.000]	[1.000,1.000]	[1.000,1.000]	[1.000,1.000]
Intermediate (30 – 69.9%)	0.928***	0.924***	0.884***	0.884***
	[0.925,0.930]	[0.922,0.927]	[0.880,0.887]	[0.880,0.888]
High (70 – 99.9%)	0.897***	0.890***	0.848***	0.857***
	[0.895,0.898]	[0.888,0.892]	[0.845,0.852]	[0.853,0.860]
Consolidated (100%)	0.840***	0.824***	0.758***	0.789***
	[0.839,0.840]	[0.823,0.825]	[0.756,0.759]	[0.787,0.791]
**Control Variables**				
Others social programs		0.972***		0.997*
		[0.970,0.974]		[0.995,1.000]
Fertility rate		0.999		1.018***
		[0.997,1.001]		[1.015,1.021]
Poverty rate (%)		0.966***		0.956***
		[0.964,0.968]		[0.953,0.959]
Proportion of individuals older than 15 years who are illiterate (%)		0.988***		1.026***
		[0.986,0.991]		[1.022,1.029]
Gini Index		1.027***		1.028***
		[1.026,1.029]		[1.025,1.031]
Piped water		1.027***		0.988***
		[1.025,1.029]		[0.984,0.991]
Households with inadequate sanitation (%)		1.019***		0.898***
		[1.017,1.021]		[0.895,0.902]
Urbanization rate		1.002		1.012***
		[0.998,1.006]		[1.007,1.017]
Hospital bed rate per 1,000 population (%)		1.002*		1.150***
		[1.000,1.004]		[1.143,1.156]
Rate of physicians per 1,000 population (%)		1		1.022***
		[0.998,1.002]		[1.019,1.025]
Year binaries	No	Yes	No	Yes
**Number of observations**	73,336	73336	73,335	73335
**Number of municipalities**	3,669	3,669	3,669	3,669

Source: Author's data analysis for 73,336 observations - 3,669 municipalities in Brazil, over 20 years (from 2000 to 2019).
Note: Data are in Rate Ratio (RR) coefficients (95% CI) unless otherwise specified. The confidence intervals are in parentheses. Time shocks are controls for specific years of economic crisis (2008, 2013, and 2015). The symbols '***' , '**' and '*' denote significance at 1%, 5%, and 10% respectively.

WEB TABLE 15. Rate Ratios from the fixed effect Poisson models for the association between overall mortality rates and Conditional Cash Transference (CCT) target coverage using categorical variables, dividing the models by poverty quartiles (heterogeneity analyses); from the period 2000-19 in Brazil.

VARIABLES	1st quartile (0 - 3.6%)	2nd quartile (3.6 - 12.9%)	3rd quartile (12.9% -29.6%)	4th quartile (29.6% - 92.3%)
**CCT target population coverage**	1	1	1	1
Low (0 – 29.9%)	[1.000,1.000]	[1.000,1.000]	[1.000,1.000]	[1.000,1.000]
Intermediate (30 – 69.9%)	0.866***	0.905***	0.948***	0.981***
	[0.841,0.892]	[0.899,0.912]	[0.942,0.954]	[0.972,0.989]
High (70 – 99.9%)	0.895***	0.871***	0.927***	0.950***
	[0.871,0.919]	[0.865,0.877]	[0.921,0.934]	[0.943,0.958]
Consolidated (100%)	0.815***	0.800***	0.908***	0.949***
	[0.799,0.831]	[0.797,0.803]	[0.902,0.915]	[0.942,0.956]
**Control Variables**				
Others social programs	0.992	0.971***	1	0.974***
	[0.983,1.002]	[0.962,0.981]	[0.993,1.007]	[0.967,0.981]
Fertility rate	1.034**	1.019***	1.004	1.020***
	[1.008,1.062]	[1.009,1.029]	[0.999,1.009]	[1.005,1.035]
Poverty rate (%)	1	1	1	1
	[1.000,1.000]	[1.000,1.000]	[0.996,1.004]	[1.000,1.000]
Proportion of individuals older than 15 years who are illiterate (%)	1.034***	1.022***	1.008**	0.99
	[1.013,1.055]	[1.011,1.033]	[1.002,1.015]	[0.972,1.008]
Gini Index	1.014***	1	0.997	1.005
	[1.008,1.021]	[0.994,1.007]	[0.989,1.005]	[0.992,1.018]
Piped water	0.955***	0.988***	0.994	1.010**
	[0.942,0.967]	[0.979,0.996]	[0.986,1.002]	[1.000,1.019]
Households with inadequate sanitation (%)	0.985***	0.989***	0.978***	0.991*
	[0.974,0.996]	[0.981,0.996]	[0.971,0.985]	[0.982,1.001]
Urbanization rate	0.837***	0.922***	0.990*	0.991
	[0.816,0.859]	[0.906,0.939]	[0.980,1.001]	[0.978,1.004]
Hospital bed rate per 1,000 population (%)	1.009*	1.004	0.996	0.987**
	[1.000,1.019]	[0.995,1.012]	[0.986,1.006]	[0.976,0.998]
Rate of physicians per 1,000 population (%)	0.955***	0.979***	1.012***	1.008**
	[0.947,0.963]	[0.971,0.987]	[1.006,1.018]	[1.001,1.016]
Year binaries	Yes	Yes	Yes	Yes
**Number of observations**	18,240	18,289	18,168	18,109
**Number of municipalities**	1,937	2,618	2,850	1,831

Source: Author's data analysis for 73,336 observations - 3,669 municipalities in Brazil, over 20 years (from 2000 to 2019).
Note: Data are in Rate Ratio (RR) coefficients (95% CI) unless otherwise specified. The confidence intervals are in parentheses. Time shocks are controls for specific years of economic crisis (2008, 2013, and 2015). The symbols '***' , '**' and '*' denote significance at 1%, 5%, and 10% respectively.

WEB TABLE 16. Rate Ratios from the fixed effect Poisson models for the association between overall hospitalization rates and Conditional Cash Transference (CCT) target coverage using categorical variables, dividing the models by poverty quartiles (heterogeneity analyses); from the period 2000-19 in Brazil.

VARIABLES	1st quartile (0 - 3.6%)	2nd quartile (3.6 - 12.9%)	3rd quartile (12.9% -29.6%)	4th quartile (29.6% - 92.3%)
**CCT target population coverage**	1	1	1	1
Low (0 – 29.9%)	[1.000,1.000]	[1.000,1.000]	[1.000,1.000]	[1.000,1.000]
Intermediate (30 – 69.9%)	0.813***	0.891***	0.948***	0.891***
	[0.780,0.847]	[0.882,0.901]	[0.940,0.956]	[0.884,0.897]
High (70 – 99.9%)	0.865***	0.876***	0.933***	0.857***
	[0.831,0.900]	[0.866,0.886]	[0.924,0.942]	[0.851,0.863]
Consolidated (100%)	0.778***	0.819***	0.927***	0.792***
	[0.757,0.800]	[0.813,0.826]	[0.920,0.935]	[0.787,0.797]
**Control Variables**				
Others social programs	1.012***	1.022***	1.004	1.003

VARIABLES	1st quartile (0 - 3.6%)	2nd quartile (3.6 - 12.9%)	3rd quartile (12.9% -29.6%)	4th quartile (29.6% - 92.3%)
	[1.004,1.019]	[1.014,1.030]	[0.997,1.011]	[0.997,1.009]
Fertility rate	0.951***	1.004	1.057***	1.135***
	[0.940,0.963]	[0.996,1.011]	[1.050,1.064]	[1.119,1.152]
Poverty rate (%)	1	1	1	1
	[1.000,1.000]	[1.000,1.000]	[1.000,1.000]	[1.000,1.000]
Proportion of individuals older than 15 years who are illiterate (%)	1.011*	1.015***	1.051***	1.038***
	[0.999,1.023]	[1.007,1.024]	[1.042,1.060]	[1.020,1.057]
Gini Index	1.079***	1.059***	1.017***	1.065***
	[1.070,1.088]	[1.052,1.066]	[1.010,1.025]	[1.054,1.076]
Piped water	1.014**	0.972***	0.996	0.948***
	[1.003,1.025]	[0.962,0.982]	[0.986,1.007]	[0.940,0.956]
Households with inadequate sanitation (%)	0.952***	0.861***	0.950***	0.968***
	[0.935,0.969]	[0.852,0.871]	[0.941,0.960]	[0.961,0.975]
Urbanization rate	1.013*	1.015**	0.974***	0.999
	[0.998,1.029]	[1.001,1.029]	[0.963,0.986]	[0.988,1.011]
Hospital bed rate per 1,000 population (%)	1.098***	1.139***	1.058***	1.103***
	[1.082,1.113]	[1.122,1.155]	[1.042,1.074]	[1.091,1.115]
Rate of physicians per 1,000 population (%)	1.014***	1.014***	1.014***	1.015***
	[1.007,1.020]	[1.008,1.021]	[1.008,1.021]	[1.009,1.021]
Year binaries	Yes	Yes	Yes	Yes
**Number of observations**	18240	18288	18168	18109
**Number of municipalities**	1,937	2,618	2,850	1,831

Source: Author's data analysis for 73,336 observations - 3,669 municipalities in Brazil, over 20 years (from 2000 to 2019).
Note: Data are in Rate Ratio (RR) coefficients (95% CI) unless otherwise specified. The confidence intervals are in parentheses. Time shocks are controls for specific years of economic crisis (2008, 2013, and 2015). The symbols '***' , '**' and '*' denote significance at 1%, 5%, and 10% respectively.

WEB TABLE 17. Rate Ratios from the fixed effect Poisson models for the association between under-five mortality rates and Conditional Cash Transference (CCT) target coverage using categorical variables, by different causes of deaths (negative control); from the period 2000-19, in Brazil.

VARIABLES	Diarrhoeal diseases	Malnutrition	Tuberculosis	Lower respiratory infections	HIV/AIDS	External causes
**CCT target population coverage**	1	1	1	1	1	1
Low (0 – 29.9%)	[1.000,1.000]	[1.000,1.000]	[1.000,1.000]	[1.000,1.000]	[1.000,1.000]	[1.000,1.000]
Intermediate (30 – 69.9%)	0.881***	0.772***	0.853	0.917***	0.655***	140934.4
	[0.837,0.928]	[0.720,0.828]	[0.574,1.268]	[0.877,0.958]	[0.519,0.825]	[0.000,.]
High (70 – 99.9%)	0.717***	0.552***	0.721	0.875***	0.566***	102470.4
	[0.680,0.757]	[0.511,0.596]	[0.479,1.087]	[0.838,0.913]	[0.450,0.714]	[0.000,.]
Consolidated (100%)	0.388***	0.293***	0.552***	0.679***	0.269***	88886.5
	[0.371,0.405]	[0.275,0.312]	[0.419,0.728]	[0.660,0.699]	[0.234,0.310]	[0.000,.]
Control Variables	Yes	Yes	Yes	Yes	Yes	Yes
Year binaries	Yes	Yes	Yes	Yes	Yes	Yes
**Number of observations**	45,345	38,344	45,55	53,095	10,435	7,057
**Number of municipalities**	2,452	2,046	234	2,971	531	522

Note: Data are in Rate Ratio (RR) coefficients (95% CI) unless otherwise specified. The confidence intervals are in parentheses. Time shocks are controls for specific years of economic crisis (2008, 2013, and 2015) and for specific years of changes in CCT programs (2003, 2004, and 2014). The symbols '***' , '**' and '*' denote significance at 1%, 5%, and 10% respectively.

WEB TABLE 18. Rate Ratios from the fixed effect Poisson models for the association between mortality rates and Conditional Cash Transference (CCT) target coverage using categorical variables, by different age groups; from the period 2000-19, in Brazil.

VARIABLES	MORTALITY BY AGE GROUPS											
	Under-1 years old	1-4 years old	5-9 years old	10-14 years old	15-19 years old	20-29 years old	30-39 years old	40-49 years old	50-59 years old	60-69 years old	70-79 years old	80 years or more
**CCT target population coverage**	1	1	1	1	1	1	1	1	1	1	1	1
Low (0 – 29.9%)	[1.000,1.000]	[1.000,1.000]	[1.000,1.000]	[1.000,1.000]	[1.000,1.000]	[1.000,1.000]	[1.000,1.000]	[1.000,1.000]	[1.000,1.000]	[1.000,1.000]	[1.000,1.000]	[1.000,1.000]
Intermediate (30 – 69.9%)	0.893***	0.894***	0.914***	0.941***	0.934**	0.904***	0.907***	0.933***	0.945***	0.944***	0.968***	0.984***
	[0.877,0.908]	[0.869,0.920]	[0.881,0.949]	[0.904,0.980]	[0.873,0.999]	[0.857,0.953]	[0.877,0.939]	[0.915,0.951]	[0.934,0.957]	[0.932,0.955]	[0.958,0.978]	[0.972,0.996]
High (70 – 99.9%)	0.848***	0.820***	0.927***	0.927***	0.886***	0.857***	0.862***	0.896***	0.919***	0.911***	0.948***	0.969***
	[0.831,0.865]	[0.791,0.849]	[0.886,0.971]	[0.894,0.962]	[0.813,0.966]	[0.809,0.908]	[0.835,0.891]	[0.879,0.913]	[0.911,0.927]	[0.902,0.920]	[0.937,0.958]	[0.963,0.976]
Consolidated (100%)	0.734***	0.720***	0.833***	0.860***	0.876**	0.836***	0.795***	0.816***	0.853***	0.837***	0.886***	0.979***
	[0.721,0.748]	[0.701,0.739]	[0.806,0.860]	[0.823,0.898]	[0.787,0.976]	[0.763,0.917]	[0.751,0.842]	[0.790,0.842]	[0.840,0.866]	[0.827,0.847]	[0.876,0.896]	[0.966,0.992]
**Control Variables**												
Others social programs	1.076***	1.089***	0.987	0.978	0.926***	0.934***	0.950***	0.965***	0.976***	0.982***	0.986***	0.978***
	[1.053,1.099]	[1.056,1.123]	[0.955,1.021]	[0.947,1.011]	[0.889,0.965]	[0.906,0.962]	[0.931,0.970]	[0.953,0.977]	[0.967,0.985]	[0.973,0.991]	[0.978,0.994]	[0.971,0.985]
Fertility rate	1.094***	1.070***	1.083***	1.015	0.888***	0.946**	0.993	1	1.011**	1.022***	1.010**	0.983***
	[1.069,1.120]	[1.035,1.106]	[1.044,1.123]	[0.983,1.049]	[0.841,0.939]	[0.907,0.987]	[0.971,1.017]	[0.987,1.014]	[1.001,1.022]	[1.012,1.032]	[1.000,1.019]	[0.974,0.991]
Poverty rate (%)	1.02	1.025	1.034*	0.954***	0.811***	0.859***	0.923***	0.967***	0.994	0.982***	0.982***	0.964***
	[0.995,1.046]	[0.993,1.058]	[0.997,1.073]	[0.922,0.987]	[0.746,0.882]	[0.806,0.915]	[0.892,0.954]	[0.949,0.985]	[0.981,1.008]	[0.970,0.993]	[0.971,0.993]	[0.953,0.975]
Proportion of individuals older than 15 years who are illiterate (%)	0.982	1.013	1.03	0.996	0.999	1.013	0.992	1.008	1.016**	0.994	1.001	0.986**
	[0.947,1.019]	[0.972,1.055]	[0.981,1.082]	[0.955,1.039]	[0.942,1.059]	[0.964,1.063]	[0.964,1.021]	[0.991,1.025]	[1.001,1.031]	[0.980,1.009]	[0.990,1.012]	[0.974,0.997]
Gini Index	1.040***	1.038***	1.055***	1.015	0.986	1.002	1.025**	1.028***	1.025***	1.032***	1.025***	1
	[1.022,1.059]	[1.010,1.067]	[1.019,1.093]	[0.985,1.047]	[0.941,1.034]	[0.968,1.039]	[1.002,1.049]	[1.012,1.044]	[1.014,1.035]	[1.023,1.042]	[1.016,1.034]	[0.993,1.007]
Piped water	0.973	0.956**	1.03	1.039**	1.095***	1.066***	1.037***	1.024***	1.020***	1.022***	1.010*	1.020***
	[0.941,1.006]	[0.916,0.997]	[0.989,1.073]	[1.002,1.078]	[1.042,1.150]	[1.030,1.103]	[1.016,1.059]	[1.006,1.042]	[1.008,1.032]	[1.011,1.032]	[1.000,1.021]	[1.011,1.029]
Households with inadequate sanitation (%)	0.924***	0.903***	0.961**	0.986	1.074**	1.081**	1.023	0.996	0.979***	0.997	0.99	1.057***
	[0.904,0.945]	[0.878,0.928]	[0.930,0.993]	[0.953,1.020]	[1.000,1.154]	[1.019,1.148]	[0.987,1.060]	[0.976,1.015]	[0.965,0.994]	[0.980,1.015]	[0.977,1.003]	[1.046,1.069]
Urbanization rate	0.941***	0.894***	0.979	0.972	0.988	0.992	1.005	1.017	0.994	0.997	0.996	1.01
	[0.914,0.969]	[0.853,0.938]	[0.920,1.041]	[0.923,1.024]	[0.931,1.050]	[0.958,1.026]	[0.977,1.034]	[0.997,1.037]	[0.976,1.013]	[0.981,1.013]	[0.983,1.009]	[0.998,1.022]
Hospital bed rate per 1,000 population (%)	1.008	1.024	1.016	1.008	0.950**	1.002	1.01	1.027***	1.020***	0.998	1.002	0.983***
	[0.984,1.033]	[0.988,1.061]	[0.975,1.058]	[0.964,1.055]	[0.907,0.996]	[0.973,1.033]	[0.992,1.028]	[1.007,1.047]	[1.005,1.036]	[0.985,1.011]	[0.991,1.014]	[0.973,0.993]
Rate of physicians per 1,000 population (%)	0.991	0.957***	0.992	1.013	1.003	1.003	0.997	0.988	0.985*	0.994	0.990*	1.014***

VARIABLES	MORTALITY BY AGE GROUPS											
	**Under-1 years old**	**1-4 years old**	**5-9 years old**	**10-14 years old**	**15-19 years old**	**20-29 years old**	**30-39 years old**	**40-49 years old**	**50-59 years old**	**60-69 years old**	**70-79 years old**	**80 years or more**
	[0.974,1.008]	[0.930,0.984]	[0.958,1.027]	[0.982,1.045]	[0.963,1.046]	[0.973,1.034]	[0.975,1.019]	[0.972,1.005]	[0.970,1.001]	[0.977,1.011]	[0.981,1.000]	[1.008,1.021]
**Time trend control**												
2008	1.076***	1.097***	1.131***	1.042***	0.972*	1.011	1.058***	1.087***	1.064***	1.068***	1.046***	0.971***
	[1.062,1.090]	[1.068,1.127]	[1.091,1.173]	[1.011,1.075]	[0.943,1.002]	[0.993,1.029]	[1.046,1.071]	[1.075,1.099]	[1.056,1.071]	[1.061,1.075]	[1.039,1.053]	[0.965,0.977]
2013	0.981***	0.934***	0.989	1.019	1.059***	0.995	1.009	1.016***	1.009***	0.988***	1.004**	1.009***
	[0.967,0.995]	[0.906,0.962]	[0.954,1.024]	[0.987,1.051]	[1.021,1.099]	[0.970,1.021]	[0.997,1.021]	[1.005,1.027]	[1.003,1.015]	[0.982,0.994]	[1.000,1.008]	[1.004,1.013]
2015	0.929***	0.821***	0.877***	0.960**	1.034**	0.969***	0.961***	0.960***	0.974***	0.970***	0.987***	1.020***
	[0.917,0.941]	[0.796,0.848]	[0.843,0.913]	[0.928,0.992]	[1.007,1.063]	[0.951,0.988]	[0.946,0.976]	[0.947,0.973]	[0.966,0.981]	[0.963,0.976]	[0.980,0.994]	[1.014,1.025]
**Number of observations**	73,296	70,434	68,178	69,897	73,036	73,336	73,336	73,336	73,336	73,336	73,336	73,336
**Number of municipalities**	3,667	3,524	3,441	3,497	3,654	3,669	3,669	3.669	3,669	3,669	3,669	3.,667

Note: Data are in Rate Ratio (RR) coefficients (95% CI) unless otherwise specified. The confidence intervals are in parentheses. Time shocks are controls for specific years of economic crisis (2008, 2013, and 2015) and for specific years of changes in CCT programs (2003, 2004, and 2014). The symbols '***' , '**' and '*' denote significance at 1%, 5%, and 10% respectively.

WEB TABLE 19. Rate Ratios from the fixed effect Poisson models for the association between overall mortality rates and Conditional Cash Transference (CCT) target coverage using categorical variables, with different specifications; from the period 2000-19 in Brazil.

VARIABLES	Original	Excluding variables		Adding variables		
		BPC coverage	Poverty	Women-led	Nurse rate	Rural households
**CCT target population coverage**	1	1	1	1	1	1
Low (0 – 29.9%)	[1.000,1.000]	[1.000,1.000]	[1.000,1.000]	[1.000,1.000]	[1.000,1.000]	[1.000,1.000]
Intermediate (30 – 69.9%)	0.924***	0.925***	0.928***	0.933***	0.929***	0.935***
	[0.912,0.937]	[0.913,0.938]	[0.916,0.941]	[0.920,0.946]	[0.917,0.942]	[0.922,0.948]
High (70 – 99.9%)	0.890***	0.891***	0.894***	0.904***	0.901***	0.907***
	[0.880,0.899]	[0.882,0.900]	[0.885,0.903]	[0.896,0.913]	[0.893,0.909]	[0.900,0.915]
Consolidated (100%)	0.824***	0.826***	0.831***	0.844***	0.846***	0.860***
	[0.807,0.842]	[0.808,0.844]	[0.813,0.849]	[0.825,0.863]	[0.825,0.866]	[0.837,0.883]
**Control Variables**						
Others social programs	0.972***			0.969***	0.965***	0.966***
	[0.964,0.981]			[0.960,0.977]	[0.956,0.973]	[0.954,0.978]
Fertility rate	0.999	0.997	0.984***	0.995	0.992*	0.993
	[0.989,1.008]	[0.987,1.007]	[0.973,0.996]	[0.986,1.005]	[0.982,1.001]	[0.982,1.004]
Poverty rate (%)	0.966***	0.966***		0.971***	0.969***	0.969***
	[0.953,0.979]	[0.953,0.979]		[0.957,0.984]	[0.956,0.983]	[0.954,0.984]
Proportion of individuals older than 15 years who are illiterate (%)	0.988	0.987*	0.981***	0.987*	0.986*	0.991
	[0.975,1.002]	[0.974,1.001]	[0.967,0.995]	[0.973,1.001]	[0.972,1.000]	[0.976,1.005]
Gini Index	1.027***	1.026***	1.026***	1.018***	1.014**	1.017**
	[1.017,1.038]	[1.015,1.037]	[1.015,1.038]	[1.006,1.029]	[1.003,1.025]	[1.003,1.031]
Piped water	1.027***	1.030***	1.030***	1.033***	1.033***	1.036***
	[1.015,1.039]	[1.018,1.042]	[1.017,1.043]	[1.021,1.045]	[1.021,1.046]	[1.022,1.051]
Households with inadequate sanitation (%)	1.019**	1.019**	1.026***	1.020**	1.024***	1.026***
	[1.004,1.034]	[1.004,1.035]	[1.009,1.043]	[1.004,1.036]	[1.008,1.040]	[1.008,1.043]
Urbanization rate	1.002	1.005	1.005	1.007	1.009	1.012
	[0.989,1.015]	[0.992,1.018]	[0.992,1.018]	[0.994,1.020]	[0.996,1.022]	[0.993,1.032]
Hospital bed rate per 1,000 population (%)	1.002	1.002	1.001	0.999	0.997	0.997
	[0.991,1.013]	[0.991,1.013]	[0.990,1.013]	[0.988,1.011]	[0.987,1.008]	[0.987,1.008]
Rate of physicians per 1,000 population (%)	1	1.001	1.001	1.001	1.007	1.009
	[0.984,1.016]	[0.985,1.018]	[0.985,1.019]	[0.983,1.019]	[0.992,1.023]	[0.991,1.027]
Women-led households (%)				0.957***	0.966***	0.960***
				[0.948,0.967]	[0.955,0.976]	[0.953,0.968]
Rate of nurses per 1,000 population (%)					0.967***	0.968***
					[0.957,0.977]	[0.957,0.981]
Rural households (%)						1.14
						[0.747,1.739]
**Time trend control**						
2008	1.048***	1.047***	1.049***	1.045***	1.040***	1.034***
	[1.042,1.054]	[1.041,1.054]	[1.042,1.055]	[1.039,1.052]	[1.032,1.048]	[1.026,1.041]
2013	0.997	0.996*	0.997	0.999	1.001	1.004
	[0.993,1.001]	[0.992,1.001]	[0.993,1.001]	[0.994,1.004]	[0.996,1.006]	[0.999,1.008]
2015	0.978***	0.978***	0.979***	0.980***	0.983***	0.987***
	[0.971,0.985]	[0.971,0.985]	[0.972,0.986]	[0.973,0.987]	[0.975,0.991]	[0.979,0.995]
**Number of observations**	73336	73336	73336	73336	73336	72316
**Number of municipalities**	3667	3667	3667	3667	3667	3667

Note: Data are in Rate Ratio (RR) coefficients (95% CI) unless otherwise specified. The confidence intervals are in parentheses. Time shocks are controls for specific years of economic crisis (2008, 2013, and 2015) and for specific years of changes in CCT programs (2003, 2004, and 2014). The symbols '***' , '**' and '*' denote significance at 1%, 5%, and 10% respectively.

WEB TABLE 20. Rate Ratios from the fixed effect Poisson models for the association between mortality rates and Conditional Cash Transference (CCT) target coverage using the Decentralized Management Index (IGDM); from the period 2000-19, in Brazil.

VARIABLES	Mortality	
	Only with IGDM	With IGDM and other controls
**CCT target population coverage**		
Low (0 – 29.9%)	1	1
	[1.000,1.000]	[1.000,1.000]
Intermediate (30 – 69.9%)	0.941***	0.938***
	[0.930,0.953]	[0.928,0.948]
High (70 – 99.9%)	0.931***	0.904***
	[0.907,0.956]	[0.881,0.927]
Consolidated (100%)	0.801***	0.758***
	[0.781,0.821]	[0.737,0.779]
**Control Variables**		
Decentralized Management Index - IGDM (%)	0.952***	0.941***
	[0.941,0.963]	[0.931,0.951]
Others social programs		0.949***
		[0.938,0.960]
Fertility rate		0.983***
		[0.971,0.996]
Poverty rate (%)		0.977***
		[0.964,0.991]
Proportion of individuals older than 15 years who are illiterate (%)		0.978**
		[0.961,0.996]
Gini Index		1.038***
		[1.024,1.053]
Piped water		1.043***
		[1.027,1.059]
Households with inadequate sanitation (%)		1.043***
		[1.022,1.063]
Urbanization rate		1.013
		[0.997,1.029]
Hospital bed rate per 1,000 population (%)		0.99
		[0.976,1.003]
Rate of physicians per 1,000 population (%)		1.009
		[0.982,1.035]
**Time trend control**		
2015	0.994	0.994
	[0.987,1.002]	[0.985,1.002]
**Number of observations**	36,669	36,669
**Number of municipalities**	3,669	3,669

Source: Author's data analysis for 73,336 observations - 3,669 municipalities in Brazil, over 20 years (from 2000 to 2019).
Note: Data are in Rate Ratio (RR) coefficients (95% CI) unless otherwise specified. The confidence intervals are in parentheses. Time shocks are controls for specific years of economic crisis (2008, 2013, and 2015). The symbols '***' , '**' and '*' denote significance at 1%, 5%, and 10% respectively.

4.5. Hospitalization and Deaths averted by CCT programs during 2000-2019

To simulate hospitalizations and deaths avoided due to CCT programs in 2000-19 period, we predicted coefficient $E(Y_{it} \mid X)$, here X represents the set of covariates including the interventions, and Y_{it} are the mortality and hospitalization rate at municipality i, in year t. Thus, the Monte Carlo methodology was used to get more accurate results compared with conventional methods such as the use of the normal distribution. It can be summarized in the following steps

1. Predict the intervention values for the retrospective period (2000-2019) initially using the same coefficients as the main model (Table 2);
2. Simulate a new Y_{it} from the Poisson distribution using the estimated parameters from the retrospective study, changing only the CCT coefficient to rescue the baseline (0% coverage) and compared with real deaths, making the difference between them and adding them up over the different years;
3. Get the predictions $E(Y_{it} \mid X)$ using the new simulated variable Y_{it}, here X represents the set of covariates including the interventions.
4. Get back to step 1.

The algorithm ended when the number of desired Monte Carlo simulations M was reached. For each outcome, 10,000 simulations were performed, chosen based on the stabilization of the estimates.

Web-Table 21 shows this simulation, in which CCT programs avoided more than 8 million hospitalizations and 700,000 deaths between 2000-19 in the hypothetical case these programs did not exist (0% coverage).

WEB-TABLE 21. Overall hospitalization and death avoided by Conditional Cash Transference (CCT) coverage during 2000-19.

	Estimate	LI	LS
Averted hospitalizations	8,176,047	4,336,139	12,280,661
Averted deaths	715,069	289,813	1,151,145

PART III – FORECASTING ANALYSIS

5. Description of the forecasting methodology

The following section provides details of the forecasting process in accordance with standard international modelling reporting guidelines (ISPOR-SMDM). The modelling approach adopted for this study was developed based on two stages.

In the first stage, a synthetic cohort of all Brazilian municipalities for the period 2024-2030 was created as an extension of a longitudinal dataset of 5,570 municipalities for 2000-2023 obtained from the sources detailed in Web Table 1. Simulated municipality-specific trends for poverty rates and the other demographic and socioeconomic variables were obtained according to economic crisis scenarios for the years 2024-2030. BFP coverage was simulated according to social protection policy response scenario options.

In the second stage, for each year and each municipality, the mortality and hospitalization rate for all the municipalities were predicted as the responses of the same multivariate fixed effects regressions described in section 3, using the forecast demographic, socioeconomic and exposure variables (BFP coverage) as input values.

6. Purpose of the forecasting and its applications

The developed model had the overall purpose of simulating the effects of socioeconomic and policy coverage changes on health outcomes in Brazil using ecologic-level data and - when available - retrospective ecologic datasets. Elements of flexibility have been introduced in the code to allow the simulation of different sets of variables and different regression models.

7. Inputs, outputs, and other parameters

7.1. Scenarios of poverty and coverage of BFP programs

In order to develop forecasting, exponential functions were used to simulate the covariates behavior for the next 7 years (2024-2030). Regarding the poverty rate, we analyzed the trajectory of the BFP eligibility criteria and simulated some changes. The BPF used two different poverty lines to identify eligible families: extreme poverty and poverty lines. In 2022, the extreme poverty line was discontinued, and the monetary cutoff for classifying a family in a poverty situation was US$2.71 per person per day at purchasing power parity (PPP), aligning closely with the World Bank's extreme poverty line of US$2.15 PPP. In contrast, other Brazilian social programs use higher poverty criteria, ranging from **¼ to ½ of the monthly minimum wage (m.w.)** equivalent to US$ 3.91 and US$ 7.8 PPP per capita per day, respectively. Thus, projections will consider a hypothetical situation where the BFP eligibility criterion is adjusted to ½ m.w.

In response to the increasing poverty rate criteria, three policy responses were considered in the main analysis:

- Expansion (Mitigation scenario): Policymakers extend the Bolsa Família Program (BFP) coverage to include all individuals who now fall below the poverty line due to the adjustment of the eligibility criterion to half of the monthly minimum wage per capita.
- Maintenance (Baseline scenario): In this scenario, policymakers maintain the same coverage proportion of the BFP relative to the population in poverty as per the previous eligibility criterion (R$ 218.00). This means that although the total number of individuals in poverty has increased due to the new eligibility threshold, the BFP coverage remains proportionally the same. Consequently, there is an absolute increase in the number of beneficiaries, but no change in the relative coverage rate.
- Reduction (Fiscal austerity scenario): Policymakers do not expand BFP coverage, keeping it on the same growth trajectory observed in past years until 2030. This scenario is based on a validated model—previously used in other studies[5,6] —which projected the effects of

ongoing fiscal austerity measures under Constitutional Amendment 95 (EC95) on BFP coverage.

The Web Figure 11 show the behavior of the poverty scenarios.

WEB FIGURE 11. Policies behavior to adjustments in the Bolsa Família Program's poverty threshold

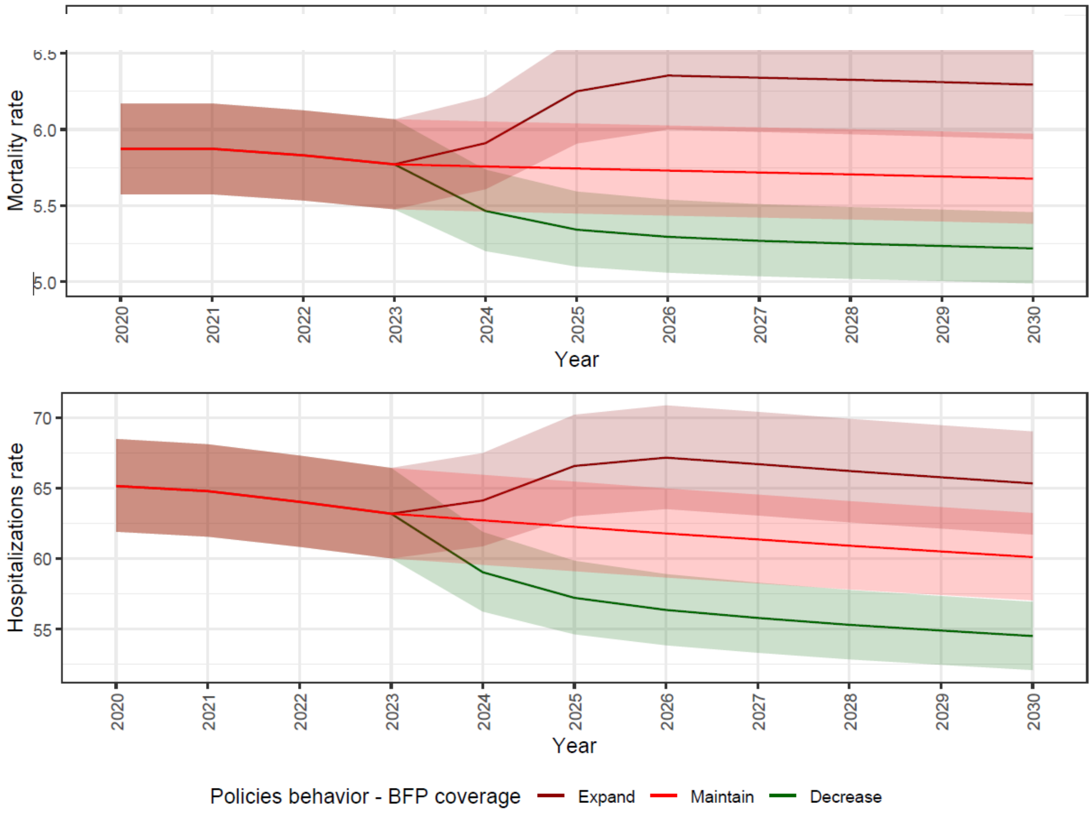

8. Prediction methodology

To generate predictions and confidence intervals for each response Y_{it}, the Monte Carlo methodology was used. This procedure allows us to get more accurate results compared with conventional methods such as the use of the normal distribution. It can be summarized in the following steps

5. Simulate the intervention values for the forecasting period (2020 -2030) using the mitigation and austerity scenarios settled in previous sections. Also simulate the control covariates using equations (2) and (3) and following their trend.
6. Simulate a new Y_{it} from the Poisson distribution using the estimated parameters from the retrospective study and the forecasted covariates.
7. Get the predictions $E(Y_{it} \mid X)$ using the new simulated variable Y_{it}, here X represents the set of covariates including the interventions.
8. Get back to step 1.

The algorithm ended when the number of desired Monte Carlo simulations M is reached. The predictions and confidence interval estimated for Y_{it} will be the mean and the percentiles 2.5% and 97.5% of the M simulations respectively. For each outcome and each scenario, 10,000 simulations were performed, allowing parameter values to vary in each simulation cycle according to their assumed underlying distribution. The number 10,000 was chosen based on the stabilization of the estimates.

9. External validation of the model.

The external validation of the model was undertaken by comparing the overall national mortality and hospitalization rates (computed for each municipality) forecasted using microsimulations, with the official Brazilian mortality and hospitalization estimates (overall) during the years 2010-2019, and estimating the linear regression and the correlation coefficients (R2) of predicted vs observed values, as shown in Web-Figures 12 and 13.

WEB FIGURE 12. Linear regression and correlation coefficient (R2) of predicted vs observed values, and trend of the simulated overall mortality rate vs the official Brazilian mortality rate estimates for the period 2010-2019.

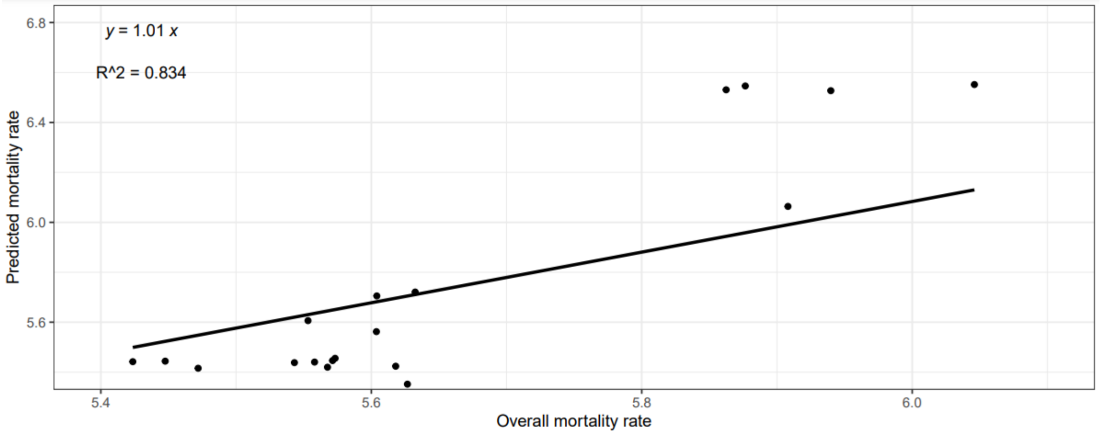

WEB FIGURE 13. Linear regression and correlation coefficient (R2) of predicted vs observed values, and trend of the simulated overall hospitalization rate vs the official Brazilian mortality rate estimates for the period 2010-2019.

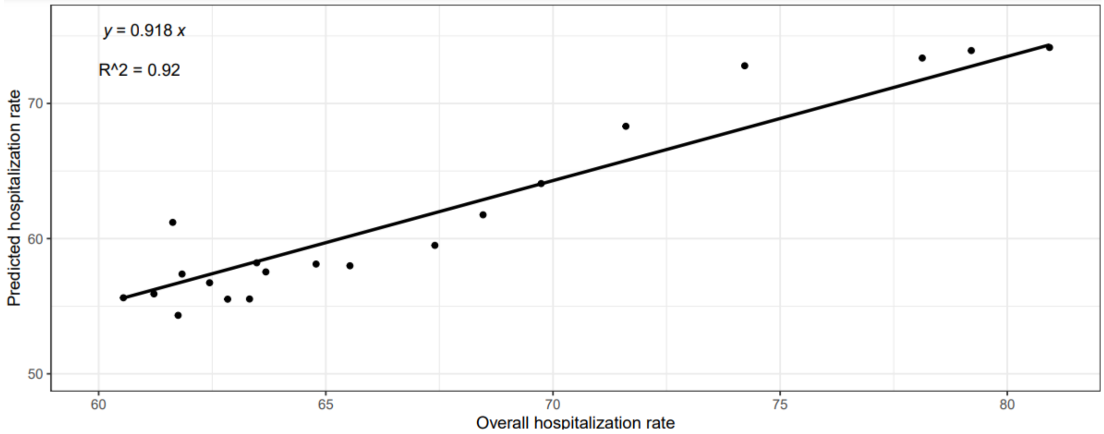

10. Sensitivity analysis

To evaluate the potential impact of new poverty lines on mortality rates, we modeled two policy response scenarios for CCT coverage: expansion and maintenance (see Web Figure 11). The findings in Web Table 22 align with those from Table 3 (see main manuscript), demonstrating that preventable deaths decline as policy conditions improve—specifically, with increased Bolsa Família Program coverage relative to the new poverty thresholds (i.e., the revised BFP eligibility criteria).

WEB TABLE 22. Rate ratio (RR) and cumulative difference in overall hospitalizations and deaths over the period 2020-2030 between alternative policy scenarios.

	HOSPITALIZATION			
	Expansion vs Baseline		Reduction vs Baseline	
Year	Rate Ratio (Number)	[95% Prediction Intervals]	Rate Ratio (Number)	[95% Prediction Intervals]
2020	0.943	0.942 – 0.943	0.923	0.882 – 0.922
2025	0.920	0.919 – 0.920	0.860	0.827 – 0.858
2030	0.907	0.907 – 0.908	0.835	0.827 – 0.832
Avoidable *hospitalization*	8,046,079	[8,023,306 – 8,068,416]	15,038,501	[15,007,706 – 15,068,079]
	**DEATHS**			
	Expansion vs Baseline		Reduction vs Baseline	
Year	Rate Ratio (Number)	[95% Prediction Intervals]	Rate Ratio (Number)	[95% Prediction Intervals]
2020	0.949	0.948 – 0.950	0.897	0.895 – 0.899
2025	0.930	0.929 – 0.932	0.831	0.828 – 0.831
2030	0.920	0.919 – 0.921	0.805	0.799 – 0.801
Avoidable deaths	683,721	[676,494 – 690,843]	1,541,591	[1,530,253 – 1,552,067]

Source: Author's data analysis for 5,570 municipalities in Brazil.
Note: Data are in Rate Ratio (RR) coefficients (95% CI) unless otherwise specified.

11. Main Limitations

The main limitation of the study is the uncertainty around the future macroeconomic scenarios in Brazil due to the current extremely unstable political and economic situation, which creates uncertainty around the forecasting of poverty rates, income, and the other independent variables. For that reason, several scenarios have been simulated in sensitivity analyses which produced comparative findings. Another limitation is that the modelling of austerity measures is focused on BFP programs as there is strong evidence that these policies confer protective effects for overall morbidity and mortality from previous studies.[22]

Our estimates of the impact of austerity measures are probably conservative as they do not reflect constraints in other areas of public spending e.g., education, housing and other welfare programs which have known impacts on poverty and health. Moreover, austerity measures recently enshrined in the constitution of Latin American countries means that public spending will only increase in line with inflation, which will not account for the demographic growth of the population, its ageing processes, and growing costs associated with new healthcare treatments and technologies.[23–25]

This is mainly due to the assumption that WB simulations of poverty increase during economic crisis already account for BFP effects, and because reliable parameters were not available at the moment of writing.

REFERENCES

1 Rasella D, Aquino R, Santos CAT, Paes-Sousa R, Barreto ML. Effect of a conditional cash transfer programme on childhood mortality: A nationwide analysis of Brazilian municipalities. *The Lancet* 2013; **382**: 57–64.

2 Ramos D, da Silva NB, Ichihara MY, *et al.* Conditional cash transfer program and child mortality: A cross-sectional analysis nested within the 100 Million Brazilian Cohort. *PLoS Med* 2021; **18**: e1003509.

3 Rasella D, Basu S, Hone T, Paes-Sousa R, Ocké-Reis CO, Millett C. Child morbidity and mortality associated with alternative policy responses to the economic crisis in Brazil: A nationwide microsimulation study. *PLoS Med* 2018; **15**: e1002570.

4 Hone T, Mirelman AJ, Rasella D, *et al.* Effect of economic recession and impact of health and social protection expenditures on adult mortality: a longitudinal analysis of 5565 Brazilian municipalities. *Lancet Glob Health* 2019; **7**: e1575–83.

5 Moncayo AL, Granizo G, Grijalva MJ, Rasella D. Strong effect of Ecuador's conditional cash transfer program on childhood mortality from poverty-related diseases: A nationwide analysis. *BMC Public Health* 2019; **19**. DOI:10.1186/s12889-019-7457-y.

6 Fernald LC, Gertler PJ, Neufeld LM. Role of cash in conditional cash transfer programmes for child health, growth, and development: an analysis of Mexico's Oportunidades. *The Lancet* 2008; **371**: 828–37.

7 Levy S. Progress against poverty: sustaining Mexico's Progresa-Oportunidades program. Brookings Institution Press, 2007 https://books.google.com/books?hl=en&lr=&id=31zD_hBNs4YC&oi=fnd&pg=PP1&dq=Progress+Against+Poverty:+Sustaining+Mexico%E2%80%99s+Progresa-Oportunidades+Program&ots=FE7IvgRtov&sig=jX5abZqFabhHhwowta5yK9TcBbc (accessed Nov 14, 2022).

8 Dávila Lárraga LG. How Does Prospera Work? 2016 https://publications.iadb.org/publications/english/viewer/How-does-Prospera-Work-Best-Practices-in-the-Implementation-of-Conditional-Cash-Transfer-Programs-in-Latin-America-and-the-Caribbean.pdf (accessed Jan 12, 2023).

9 Rasella D, Alves FJO, Rebouças P, *et al.* Long-term impact of a conditional cash transfer programme on maternal mortality: a nationwide analysis of Brazilian longitudinal data. *BMC Med* 2021; **19**. DOI:10.1186/s12916-021-01994-7.

10 Riumallo-Herl C, Aguila E. The effect of old-age pensions on health care utilization patterns and insurance uptake in Mexico. *BMJ Glob Health* 2019; **4**. DOI:10.1136/bmjgh-2019-001771.

11 Hernández Licona G et al., de la Garza T, Zamudio J, Yaschine I. El Progresa-Oportunidades-Prospera, a veinte años de su creación, 1st edn. Consejo Nacional de Evaluación de la Política de Desarrollo Social, 2019 https://www.coneval.org.mx/Evaluacion/IEPSM/Documents/Libro_POP_20.pdf (accessed Nov 16, 2022).

12 Martínez D, Borja T, Medellín N, Cueva P. ¿Cómo funciona el Bono de Desarrollo Humano? 2017 https://www.academia.edu/download/72227652/be4f0990662c39d0f1b9ef9988a9e7f224b5.pdf (accessed Nov 16, 2022).

13 de Souza PH, Osorio RG, Paiva LH, Soares S. The effects of the Bolsa Família Program on poverty and inequality: a review of the first fifteen years. 2019 https://www.econstor.eu/handle/10419/211450 (accessed Nov 16, 2022).

14 AbouZahr C, de Savigny D, Mikkelsen L, *et al.* Civil registration and vital statistics: progress in the data revolution for counting and accountability. *The Lancet* 2015; **386**: 1373–85.

15 Mikkelsen L, Phillips DE, AbouZahr C, *et al.* A global assessment of civil registration and vital statistics systems: monitoring data quality and progress. *The Lancet* 2015; **386**: 1395–406.

16 Lourenço Tavares de Andrade C, Landmann Szwarcwald C. Desigualdades sócio-espaciais da adequação das informações de nascimentos e óbitos do Ministério da Saúde, Brasil, 2000-2002 Socio-spatial inequalities in the adequacy of Ministry of Health data on births and deaths at the municipal level in Brazil. mai, 2007 http://www.datasus.

17 Rasella D, Aquino R, Barreto ML. Impact of the Family Health Program on the quality of vital information and reduction of child unattended deaths in Brazil: an ecological longitudinal study. 2010 http://www.biomedcentral.com/1471-2458/10/380.

18 Lawlor DA, Tilling K, Davey Smith G. Triangulation in aetiological epidemiology. *Int J Epidemiol* 2017; : dyw314.

19 Rasella D, Basu S, Hone T, Paes-Sousa R, Ocké-Reis CO, Millett C. Child morbidity and mortality associated with alternative policy responses to the economic crisis in Brazil: A nationwide microsimulation study. *PLoS Med* 2018; **15**. DOI:10.1371/journal.pmed.1002570.

20 Mariani, C. B. Gomes, E. C. Cenci, D. R. Queiroz RF de. Financiamento da Assistência Social no Brasil Nota Técnica de Monitoramento (2019). 2019.

21 Salata ARicardo, Ribeiro MGomes. Boletim Desigualdade nas Metrópoles (n. 09). Observatório das Metrópoles. Porto Alegre/RS, 2022.

22 Rasella D, Aquino R, Santos CAT, Paes-Sousa R, Barreto ML. Effect of a conditional cash transfer programme on childhood mortality: A nationwide analysis of Brazilian municipalities. *The Lancet* 2013; **382**. DOI:10.1016/S0140-6736(13)60715-1.

23 Paiva AB, Mesquita ACS, Jaccoud L, Passos L. [The new tax regime and its implications for social assistance policy in Brazil.] [Portuguese]. *Instituto de Pesquisa Econômica Aplicada* 2016; **27**.

24 Vieira, F. S. Benevides RPDS. [The new tax regime and its implications for social assistance policy in Brazil.] [Portuguese]. Technical Note No.27. 2016.

25 Rossi P, Dweck E. Impacts of the New Fiscal Regime on health and education Impactos. *Cad Saude Publica* 2016; **32**.
